# Plasma p-tau species are elevated in presymptomatic and symptomatic neuronal intranuclear inclusion disease

**DOI:** 10.1016/j.ebiom.2026.106127

**Published:** 2026-01-14

**Authors:** Sizhe Zhang, Bin Jiao, Yan Zeng, Qiying Sun, Xiaoyu Chen, Weiwei Zhang, Ziyu Ouyang, Qiao Xiao, Lu Zhou, Yunni Li, Ling Weng, Juan Du, Qian Xu, Yang Yang, Mengqi Zhang, Qiuming Zeng, Liangjuan Fang, Hongyu Long, Yuanyuan Xie, Si Chen, Li Feng, Qing Huang, Lili Long, Yafang Zhou, Fang Yi, Yacen Hu, Qiong Liu, Yongcheng Pan, Lin Zhou, Yulai Li, Shuo Hu, Jifeng Guo, Junling Wang, Hong Jiang, Hongwei Xu, Ranhui Duan, Beisha Tang, Yun Tian, Lu Shen

**Affiliations:** a Department of Neurology, Xiangya Hospital, Central South University, Changsha, Hunan, 410008, China; b Engineering Research Center of Hunan Province in Cognitive Impairment Disorders, Central South University, Changsha, Hunan, 410008, China; c Hunan International Scientific and Technological Cooperation Base of Neurodegenerative and Neurogenetic Diseases, Changsha, Hunan, 410008, China; d Key Laboratory of Hunan Province in Neurodegenerative Disorders, Xiangya Hospital, Central South University, Changsha, Hunan, 410008, China; e National Clinical Research Center for Geriatric Disorders, Central South University, Changsha, Hunan, 410008, China; f Department of Geriatrics, Xiangya Hospital, Central South University, Changsha, Hunan, 410008, China; g Department of Neurosurgery, Xiangya Hospital, Central South University, Changsha, Hunan, 410008, China; h Department of Radiology, Xiangya Hospital, Central South University, Changsha, Hunan, 410008, China; i Center for Medical Genetics, School of Life Sciences, Central South University, Changsha, Hunan, 410008, China; j Department of Nuclear Medicine, Xiangya Hospital, Central South University, Changsha, Hunan, 410008, China

**Keywords:** Neuronal intranuclear inclusion disease, Plasma biomarkers, Presymptomatic NIID, p-tau, tau-PET, GFAP

## Abstract

**Background:**

Neuronal intranuclear inclusion disease (NIID), caused by GGC repeat expansions in NOTCH2NLC, is a neurodegenerative disease frequently involved with cognitive impairment. Limited studies have focused on the biomarkers alteration in patients with NIID and presymptomatic NIID (preNIID) individuals. The clinical overlap between NIID and AD drives the exploration of plasma biomarker alterations in NIID and preNIID.

**Methods:**

Cohorts 1 (87 patients with NIID, 147 individuals with Alzheimer's disease [AD], and 110 healthy controls [HCs]) and 2 (26 individuals with preNIID and 26 HCs) were included. Eight plasma biomarkers including amyloid-β (Aβ) 40, Aβ42, neurofilament light (NfL), α-synuclein (α-syn), phosphorylated tau protein 181 (p-tau181), p-tau217, p-tau231, and glial fibrillary acidic protein (GFAP) were detected. Neuropsychological scores, magnetic resonance imaging measures, Aβ positron emission tomography (Aβ-PET), and tau-PET were analysed.

**Findings:**

P-tau217, p-tau231, p-tau181, α-syn, NfL, and GFAP levels were elevated in patients with NIID compared with HCs; p-tau species and GFAP were also upregulated in preNIID. P-tau species, particularly p-tau217, effectively distinguished NIID/preNIID from HCs (AUC 0.814/0.848), but failed to differentiate NIID from AD. The level of p-tau217 was associated with MMSE and FAB scores in dementia-dominant subtype, and the level of GFAP correlated to white matter volume. A tau-PET study revealed distinct tau deposition on the occipital lobe and temporal pole in NIID without Aβ pathology.

**Interpretation:**

The significant changes of p-tau levels and prominent tau deposition highlight tau pathology involvement in NIID. Elevated plasma p-tau species in preNIID/NIID indicate their potential as biomarkers for NIID.

**Funding:**

This study was supported by the 10.13039/501100001809National Natural Science Foundation of China (82394421, 82394420, 82371866, 82371434); the 10.13039/501100012166National Key R&D Program of China (2022ZD0213700); Natural Science Foundation of Hunan Province (2023JJ10097, 2025JJ40089, 2023JJ40948).

**Research in context**

**Evidence before this study**

We reviewed the available literature on the biomarkers of neuronal intranuclear inclusion disease (NIID) and found limited studies focusing on neurofilament light (NfL) and glial fibrillary acidic protein (GFAP) alterations with small sample sizes. However, the potential value of other biomarkers, including amyloid-β (Aβ) 40, Aβ42, α-synuclein (α-syn), phosphorylated tau protein 181 (p-tau181), p-tau217, and p-tau231 levels was unclear. Cerebrospinal fluid (CSF) p-tau181 has been shown to be increased in NIID, with levels comparable with those observed in Alzheimer's disease (AD). The levels of plasma p-tau species, including p-tau181, p-tau217, and p-tau231, were unclear compared to those in AD. Additionally, alterations in these biomarkers in presymptomatic NIID (preNIID) have not been evaluated.

**Added value of this study**

Our study found that p-tau217, p-tau231, p-tau181, α-syn, NfL, and GFAP levels were significantly higher in NIID than healthy controls (HCs), expanding the biomarker spectrum of NIID. P-tau species and GFAP were already upregulated in preNIID. P-tau species, particularly p-tau217, effectively distinguished NIID and preNIID from HCs, highlighting the value of p-tau in NIID diagnosis. However, they could not differentiate NIID from AD. A tau-PET study revealed distinct tau deposition in cortex in NIID, further emphasising tau pathology involvement in NIID.

**Implications of all the available evidence**

This study comprehensively elucidated the biomarker alterations in NIID. The changes in p-tau levels in preNIID and NIID provide new insights into NIID pathophysiology, and the prominent tau deposition in NIID underscores the importance of further investigating the tau deposition patterns in the future.

## Introduction

Neuronal intranuclear inclusion disease (NIID), caused by the expansion of GGC repeats within the 5'untranslated region (UTR) of the NOTCH2NLC gene,^1^, ^2^, ^3^ is a neurodegenerative disorder characterised by eosinophilic hyaline intranuclear inclusions in the central nervous system, peripheral nervous system, and visceral organs.^4^, ^5^, ^6^, ^7^ Common neurological symptoms in NIID include cognitive impairment, paroxysmal symptoms, autonomic dysfunction, movement disorders, and muscle weakness, with cognitive impairment being the most common, occurring in 49.4–92% of patients with NIID.^4^^,^^5^^,^^8^^,^^9^ NIID can be divided into four subgroups according to its main and initial manifestations.^4^^,^^8^ Among them, the dementia-dominant subtype of NIID is clinically challenging to differentiate from Alzheimer's disease (AD).^10^

Few studies have focused on neurodegenerative biomarkers for NIID. A Japanese study of 12 patients with NIID found elevated phosphorylated tau protein 181 (p-tau181) in cerebrospinal fluid (CSF), with levels similar to those in AD.^11^ However, this study did not involve investigation of blood p-tau181 and brain tau positron emission tomography/computed tomography (tau-PET/CT). Furthermore, no blood biomarker research has been done on presymptomatic NIID (preNIID), asymptomatic NOTCH2NLC mutation carriers. Given that p-tau181 is relatively specific to AD pathology,^12^ it is also crucial to investigate whether other p-tau species, such as p-tau217 and p-tau231, which have higher specificity for AD, are also elevated in patients with NIID with p-tau181.

Additionally, glial fibrillary acidic protein (GFAP) associated with astrocytes activation, as well as alpha-synuclein (α-syn) related to synuclein pathology, remain unclear in NIID. Previous autopsy studies have reported that inclusion-bearing astrocytes are more affected than neurons in adult-onset NIID.^13^^,^^14^ And over 50% of patients with NIID present movement disorder symptoms.^4^ Given the variable clinical characteristics and complex pathological alterations in NIID, exploring the biomarker spectrum is vital for understanding its pathological mechanisms.

In the present study, we aimed to measure plasma levels of Aβ40, Aβ42, neurofilament light (NfL), α-syn, p-tau181, p-tau217, p-tau231 and GFAP in two large Chinese cohorts comprising NIID (n = 87), AD (n = 147), preNIID individuals (n = 26), and healthy controls (HCs, n = 136). We compared biomarker alterations between NIID and AD to identify biomarkers that could distinguish NIID from AD. We intended to characterise early alterations in plasma biomarkers during preNIID stage. To investigate complex pathological alterations in NIID, we also performed tau-PET imaging.

## Methods

### Participants

In this cross-sectional study, we enrolled 396 participants who were divided into two independent cohorts. The cohort 1 consisted of 87 patients with NIID, 147 patients with AD, and 110 sex- and age-matched healthy controls (HCs), recruited from Xiangya Hospital, Central South University. Demographic information, including age, sex, and ethnicity, was self-reported by participants. All participants were Han Chinese. Patients with NIID were diagnosed according to the following criteria: (1) at least one of five clinical features (cognitive impairment, paroxysmal symptoms, autonomic dysfunction, movement disorders, and muscle weakness), (2) abnormal GGC repeats (>65) within NOTCH2NLC via genetic testing, and (3) p62-positive intranuclear inclusions with skin biopsy. Clinical phenotypes were determined according to patients' initial and main clinical manifestations, and were divided into the following four subtypes: dementia-dominant, movement disorder-dominant, muscle weakness-dominant and paroxysmal symptom-dominant subtypes. The diagnosis of AD was based on the US National Institute on Aging and Alzheimer's Association research framework criteria,^15^ and all the patients with AD completed the Aβ-PET/CT or CSF biomarkers (Aβ40, Aβ42, t-tau, and p-tau181) examination. In detail, the core biomarkers of CSF were measured using an enzyme-linked immunosorbent assay, including Aβ40 (EQ 6511-9601-L), Aβ42 (EQ 6521-9601-L), t-tau (EQ 6531-9601-L), and p-tau181 (EQ 6591-9601-L) (EUROIMMUN, Germany). The cut-off values were determined according to the manufacturer's instructions, participants with Aβ42 < 550 pg/mL or Aβ42 among 551–650 pg/mL along with Aβ42/40 ≤ 0.01 were classified as A+, and participants with p-tau181 ≥ 61 pg/mL were classified as T+. The patients of NIID and AD meeting the diagnostic criteria mentioned above were selected for inclusion (Table S1). The HCs in cohort 1 were recruited from a two-year follow-up community cohort and showed no cognitive decline or other neurological diseases over the follow-up period.

Cohort 2 included 26 preNIID individuals and 26 sex- and age-matched HCs. Individuals with preNIID were screened out from the NIID pedigrees, and were defined by the expanded GGC repeats along with the absence of disease-related signs or clinical symptoms under comprehensive evaluation by at least two neurologists. Notably, the HCs in cohort 2 were from the NIID pedigrees with a normal GGC repeat within the NOTCH2NLC gene.

This study was approved by the ethical review committee of Xiangya Hospital, Central South University (No. 202202050549), and was conducted according to the tenets of the Declaration of Helsinki. All the participants provided written informed consent.

### Plasma biomarkers collection and detection

Venous blood samples were collected into tubes containing ethylenediaminetetraacetic acid. The blood was centrifuged at 2000×*g* for 10 min at 4 °C. The obtained plasma was divided into 400 μL aliquots and frozen at −80 °C. None of the samples underwent freeze–thaw cycle. Plasma Aβ40, Aβ42, NfL, α-syn, p-tau181, p-tau217, p-tau231, and GFAP levels were quantified by commercially available Single Molecular Immunity Detection kits based on the AST-Sc-Lite (A fully-auto single-molecule detection machine supplied by Suzhou AstraBio Technology Co., Ltd.), and all the tests were performed according to the manufacturer's instructions. A detailed description of the AST-Sc-Lite detection method can be found in the Supplementary Methods (Figure S1, Tables S2 and S3).

### Neuropsychological assessments

The Scales for Outcomes in Parkinson's Disease-Autonomic Questionnaire (SCOPA-AUT) was used to assess autonomic function in patients with NIID. Several scales, including the Mini-Mental State Examination (MMSE), Montreal Cognitive Assessment (MoCA), and Frontal Assessment Battery (FAB), were used to assess cognitive function. The Neuropsychiatric Inventory (NPI) was used to assess mental health problems. The Activities of Daily Living (ADL) scale was used to assess daily function.

### Magnetic resonance imaging (MRI) data acquisition and analysis

Fifty-one patients with NIID underwent brain MRI using a 3.0T MRI scanner with a 64-channel head/neck coil (Prisma, Siemens, Germany). The three-dimensional T1-weighted images scan (3D-T1WI, Sagittal, repetition time TR = 2300 ms, echo time TE = 2.98 ms, inversion time TI = 900 ms, flip angle FA = 9°, 176 slices, slice thickness = 1 mm, data matrix = 248 × 256, field of view FOV = 248 × 256 mm^2^), T2-weighted-fluid-attenuated inversion recovery scan (T2-FLAIR, Sagittal, TR = 9000 ms, TE = 83 ms, TI = 2500 ms, flip angle FA = 150°, 25 slices, slice thickness = 5 mm, data matrix = 217 × 320, field of view FOV = 213 × 220 mm^2^), and diffusion weighted imaging (DWI, Sagittal, TR = 3360 ms, TE1 = 55 ms, TE2 = 93 ms, flip angle [FA] = 180°, 25 slices, slice thickness = 5 mm, data matrix = 192 × 192, field of view [FOV] = 220 × 220 mm^2^) were acquired.

Statistical Parametric Mapping 12 (SPM12, MatLab v. 2021a,MathWorks) was used to calculate the gray matter volume (GMV), white matter volume (WMV), CSF volume (CSFV), total intracranial volume (TIV), cortical thickness, and lesion volume as previously reported.^16^ For volume Estimation of GMV, WMV, CSFV, and TIV, regions of interest (ROIs) were defined for various brain areas, and the voxels within each ROI were summed as the volume of the ROI. The GMV, WMV, and CSFV were summed to obtain TIV values. For cortical thickness analysis, the cortical surface of the brain was reconstructed, and the cortical thickness was determined as the distance between the inner and outer surfaces. For lesion volume calculation, Lesion Growth Analyzer (LGA, https://www.statistical-modeling.de/lst.html) tool was used to detect and quantify white matter hyperintensities, which are areas of increased signal intensity on T2-FLAIR images.

### PET/CT image acquisition and processing

PET/CT imaging was performed using a GE Healthcare PET/CT system to assess key biomarkers, including Aβ deposition, tau pathology, and cerebral glucose metabolism. Before the imaging procedures, all therapeutic medications were discontinued for a minimum 12-h washout period. For Aβ detection, intravenous injection of AV45 tracer was followed by a 60-min uptake period before image acquisition. Tau pathology was evaluated using the second-generation tau-specific radiotracer 18F-MK6240, with PET scanning initiated 70 min post-injection and continued for 20 min. The radiotracers 18F-fluorodeoxyglucose (18F-FDG) were administered to evaluate cerebral glucose metabolism. For 18F-FDG imaging, after intravenous tracer injection, a 50-min uptake period preceded a 10-min PET acquisition to capture metabolic activity. The visual assessment identified abnormal tracer uptake patterns deviating from physiological distribution when compared with adjacent background activity.

Image preprocessing was conducted using Statistical Parametric Mapping (SPM) 12. For each subject, the T1-weighted MRI and 18F-MK6240 PET images were first coregistered using a six-parameter rigid-body transformation. The T1-weighted images were then spatially normalised to a standard template via the unified segmentation approach, and the same transformation parameters were applied to the coregistered PET images to ensure spatial normalisation. Standardised uptake value ratio (SUVR) was calculated by normalising SUV values to the inferior cerebellar cortex. A voxel-wise analysis was performed, using a threshold of p < 0.05 and a cluster extent threshold (k) of 100 voxels. Differences in voxel between the NIID group (n = 10) and matched HCs (n = 15) were assessed with an independent two-sample t-test.

### Statistical analyses

Statistical analyses were conducted using the software program R version 4.2.0 (R Foundation for Statistical Computing, Vienna, Austria). Statistical significance was set at *p* < 0.05. Categorical and continuous variables were presented as frequencies with percentages and medians with interquartile ranges, respectively. Demographic and clinical characteristics were compared between the different groups using the Mann–Whitney U test or Kruskal–Wallis H test. Biomarker diagnostic performance was evaluated using receiver operating characteristic (ROC) curves and area under curve (AUC) values. The optimal cut-off value was determined by identifying the maximal Youden index (sensitivity + specificity–1). Multivariate binary logistic regression models predicted disease presence using the AUC calculated from the predicted probabilities. Correlation analyses were performed using Spearman correlation coefficients.

Role of Funders: The funding sources had no role in study design, data collection, data analyses, interpretation, or writing of report.

## Results

### Clinical characteristics of participants

The demographic characteristics of Cohort 1 are shown in Table 1. The age at onset (AAO) of NIID was slightly lower than that of patients with AD, and the median disease duration was longer than that of AD. The proportion of the dementia-dominant subtype (n = 34) was 39.1% in the NIID group, followed by the other three subtypes including the movement disorder-dominant subtype (30/87, 34.5%), paroxysmal symptom-dominant subtype (14/87, 16.1%), and muscle weakness-dominant subtype (9/87, 10.3%). The cognitive deficits reflected by the MMSE scores were more severe in the AD group than in the NIID group. In cohort 2, 26 individuals in the preNIID stage and 26 non-mutation carriers were included; their demographic details are summarised in Table 2.

**Table 1:** Demographic characteristics of enrolled patients with NIID and AD and HCs in cohort 1.

	HCs (n = 110)	NIID (n = 87)	AD (n = 147)	p.overall	p.control vs NIID	p.control vs AD	p.NIID vs AD
Sex				0.989	1.000	1.000	1.000
Female	63 (57.27%)	49 (56.32%)	83 (56.46%)				
Male	47 (42.73%)	38 (43.68%)	64 (43.54%)				
Age	65.00 [60.25; 68.00]	64.00 [58.00; 67.00]	63.00 [55.50; 69.00]	0.055	0.058	0.087	0.817
AAO	–	57.00 [50.00; 63.00]	59.00 [53.00; 66.00]	0.018∗	–	–	0.018∗
Duration	–	5.00 [3.00; 8.00]	2.00 [2.00; 4.00]	<0.001∗	–	–	<0.001∗
MMSE	29.00 [29.00; 30.00]	23.50 [18.00; 27.00]	15.00 [7.00; 20.00]	<0.001∗	<0.001∗	<0.001∗	<0.001∗
APOE	20 (18.2%)	14 (16.1%)	63 (42.9%)	<0.001∗	0.845	<0.001∗	<0.001∗
Aβ40	340.78 [256.59; 406.88]	297.63 [230.74; 358.19]	293.11 [243.86; 378.58]	0.060	0.065	0.065	0.877
Aβ42	70.25 [48.77; 99.34]	66.29 [44.96; 110.60]	66.03 [48.60; 89.03]	0.734	0.806	0.806	0.941
NfL	41.53 [24.82; 62.48]	76.28 [39.01; 132.00]	50.07 [32.10; 87.12]	<0.001∗	<0.001∗	0.005∗	0.018∗
α-syn	3091.16 [1908.32; 5457.04]	6545.35 [3290.98; 9320.25]	6026.65 [3582.55; 8744.55]	<0.001∗	<0.001∗	<0.001∗	0.574
p-tau181	7.05 [4.08; 9.55]	10.51 [7.69; 14.12]	10.99 [7.43; 19.53]	<0.001∗	<0.001∗	<0.001∗	0.438
p-tau217	3.58 [3.05; 4.18]	5.39 [4.10; 7.71]	5.86 [4.22; 7.56]	<0.001∗	<0.001∗	<0.001∗	0.682
p-tau231	8.00 [6.10; 10.09]	14.69 [9.18; 31.23]	14.15 [7.61; 27.80]	<0.001∗	<0.001∗	<0.001∗	0.338
GFAP	16.10 [12.78; 23.26]	26.14 [11.94; 41.37]	46.14 [29.97; 63.11]	<0.001∗	0.018∗	<0.001∗	<0.001∗

Notes: p.overall represents the p value for the overall comparison of the three groups (NIID, AD, and HCs) using the Kruskal–Wallis H test; p.control vs NIID, p.control vs AD, and p.NIID vs AD were acquired through Benjamini & Hochberg adjustment with Kruskal–Wallis H test for pairwise comparisons.

Abbreviations: NIID, neuronal intranuclear inclusion disease; AD, Alzheimer's disease; HCs, healthy controls; AAO, age at onset; MMSE, Mini-Mental State Examination; Aβ, amyloid beta; NfL, neurofilament light chain; α-syn, α-synuclein; p-tau, phosphorylated tau protein; GFAP, glial fibrillary acidic protein.

∗p < 0.05.

**Table 2:** Baseline characteristics of enrolled preNIID individuals and HCs in cohort 2.

	HCs (n = 26)	preNIID (n = 26)	p value
Sex			1.000
Female	18 (69.23%)	17 (65.38%)	
Male	8 (30.77%)	9 (34.62%)	
Age	37.50 [35.00; 39.75]	36.50 [32.50; 41.50]	0.721
Aβ40	239.44 [207.93; 286.54]	262.40 [206.60; 293.05]	0.877
Aβ42	56.38 [41.57; 71.13]	71.19 [53.29; 95.47]	0.055
NfL	27.54 [13.53; 37.59]	34.70 [17.29; 56.39]	0.280
α-syn	3058.39 [2339.08; 6139.86]	4769.10 [1896.45; 9436.16]	0.458
p-tau181	6.48 [4.72; 7.82]	9.04 [6.01; 11.28]	0.021∗
p-tau217	3.29 [2.59; 3.89]	5.23 [3.88; 7.10]	<0.001∗
p-tau231	5.99 [1.71; 14.50]	13.55 [9.28; 19.54]	0.005∗
GFAP	8.41 [3.48; 15.01]	23.11 [15.73; 38.14]	<0.001∗

Notes: p value was obtained through the Mann–Whitney U test.

Abbreviations: preNIID, presymptomatic NIID; HCs, healthy controls; Aβ, amyloid beta; NfL, neurofilament light chain; α-syn, α-synuclein; p-tau, phosphorylated tau protein; GFAP, glial fibrillary acidic protein.

∗p < 0.05.

### Plasma NfL, α-syn, p-tau, and GFAP were significantly increased in patients with NIID

**Fig. 1:**
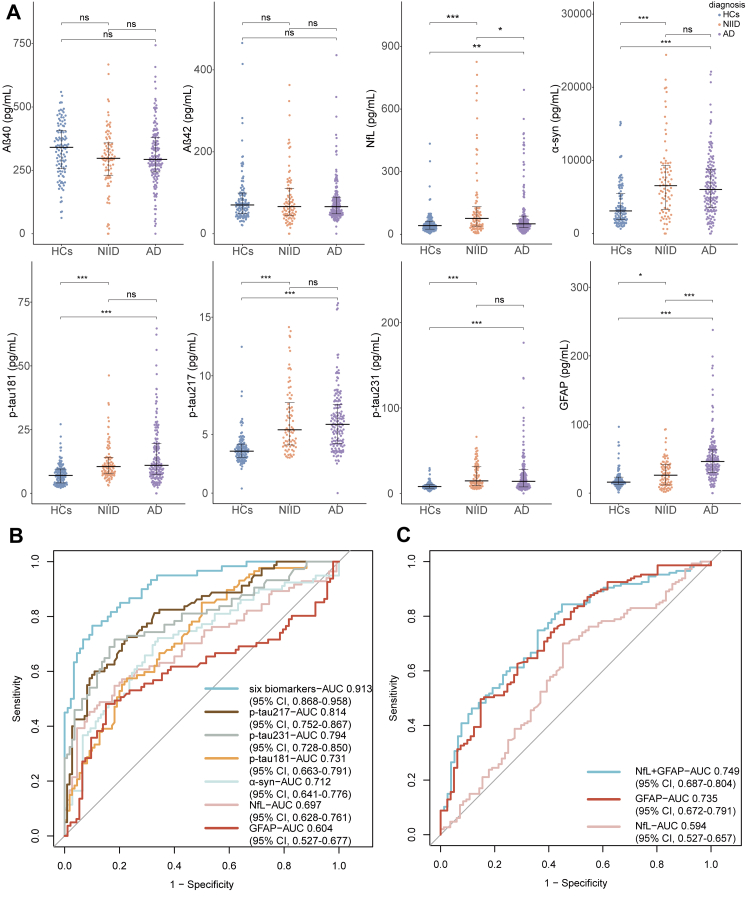
**Comparison and diagnosis performances of plasma biomarkers between NIID, AD, and HCs in cohort 1.** (A) NfL, α-syn, p-tau181, p-tau217, p-tau231, and GFAP levels showed significant differences between NIID (n = 87) and HCs (n = 110) and AD (n = 147) and HCs (n = 110). Moreover, the p-tau181, p-tau217, and p-tau231 were consistent in AD and NIID groups. The NfL was higher in NIID than in AD, whereas the GFAP was higher in the AD group. (B) The p-tau217 showed the highest AUC (0.814, 95% CI 0.752–0.867) for discriminating NIID from HCs, followed by p-tau231, p-tau181, α-syn, NfL, and GFAP levels. A combination of these six significantly different biomarkers achieved an AUC of 0.913 (95% CI 0.868–0.958) in distinguishing NIID from HCs. (C) GFAP showed better performance than NfL, with an AUC of 0.735 (95% CI 0.672–0.791). A combination of GFAP and NfL could better discriminate NIID from AD. Benjamini & Hochberg adjusted Kruskal–Wallis H test was used for pairwise comparisons. Area under the curve (AUC) and confidence intervals are shown in the figure. Multivariate binary logistic regression models predicted disease presence using the AUC calculated from the predicted probabilities. NIID, neuronal intranuclear inclusion disease; AD, Alzheimer's disease; HCs, healthy controls; NfL, neurofilament light chain; α-syn, α-synuclein; p-tau, phosphorylated tau protein; GFAP, glial fibrillary acidic protein (∗p < 0.05, ∗∗p < 0.01, ∗∗∗p < 0.001).

In cohort 1, plasma NfL, α-syn, p-tau181, p-tau217, p-tau231, and GFAP levels were significantly higher in patients with NIID and AD. In the comparison of NIID and AD, p-tau181, p-tau217, p-tau231 and α-syn were comparable between NIID and AD, whereas NfL was higher in patients with NIID compared to patients with AD, and GFAP was statistically lower in NIID than in AD (Fig. 1A). P-tau/Aβ42 demonstrated significant differences between NIID and HCs, and between AD and HCs. However, no difference was observed in p-tau/Aβ42 between NIID and AD (Figure S2). We examined whether plasma biomarker levels in NIID correlated with age at onset (AAO) (Figure S3), disease duration (Figure S4) and GGC repeats number (Figure S5). Only GFAP showed a positive correlation with AAO in NIID. The phenotypic information is presented in Table S4. No significant biomarkers were identified among the four NIID subtypes (Table S4, Figure S6).

### Diagnosis accuracy of plasma biomarkers in NIID

Next, we compared the predictive power of these six altered plasma biomarkers to distinguish patients with NIID from HCs. The AUCs of altered biomarkers were 0.814 (95% confidence interval [CI], 0.752–0.867) for p-tau217 at a cut-off value of 4.19 pg/mL, 0.794 (95% CI, 0.728–0.850) for p-tau231 at a cut-off value of 10.66 pg/mL, 0.731 (95% CI, 0.663–0.791) for p-tau181 at a cut-off value of 6.91 pg/mL, 0.712 (95% CI, 0.641–0.776) for α-syn at a cut-off value of 3797.9 pg/mL, 0.697 (95% CI, 0.628–0.761) for NfL at a cut-off value of 69.63 pg/mL, and 0.604 (95% CI, 0.527–0.677) for GFAP at a cut-off value of 28.00 pg/mL (Fig. 1B). Interestingly, p-tau217 showed the best performance in distinguishing NIID from HCs. The combination of these six biomarkers demonstrated the highest diagnostic value, with an AUC of 0.913 (95% CI, 0.868–0.958).

GFAP also showed value in distinguishing NIID from AD with an AUC of 0.735 (95% CI, 0.672–0.791) at a cut-off value of 46.33 pg/mL. The combination of NfL and GFAP improved the performance in distinguishing NIID from AD, with an AUC of 0.749 (95% CI, 0.687–0.804) (Fig. 1C). However, p-tau species were difficult in distinguishing NIID from AD.

### Correlation analysis between plasma biomarkers and neuropsychological scores

**Fig. 2:**
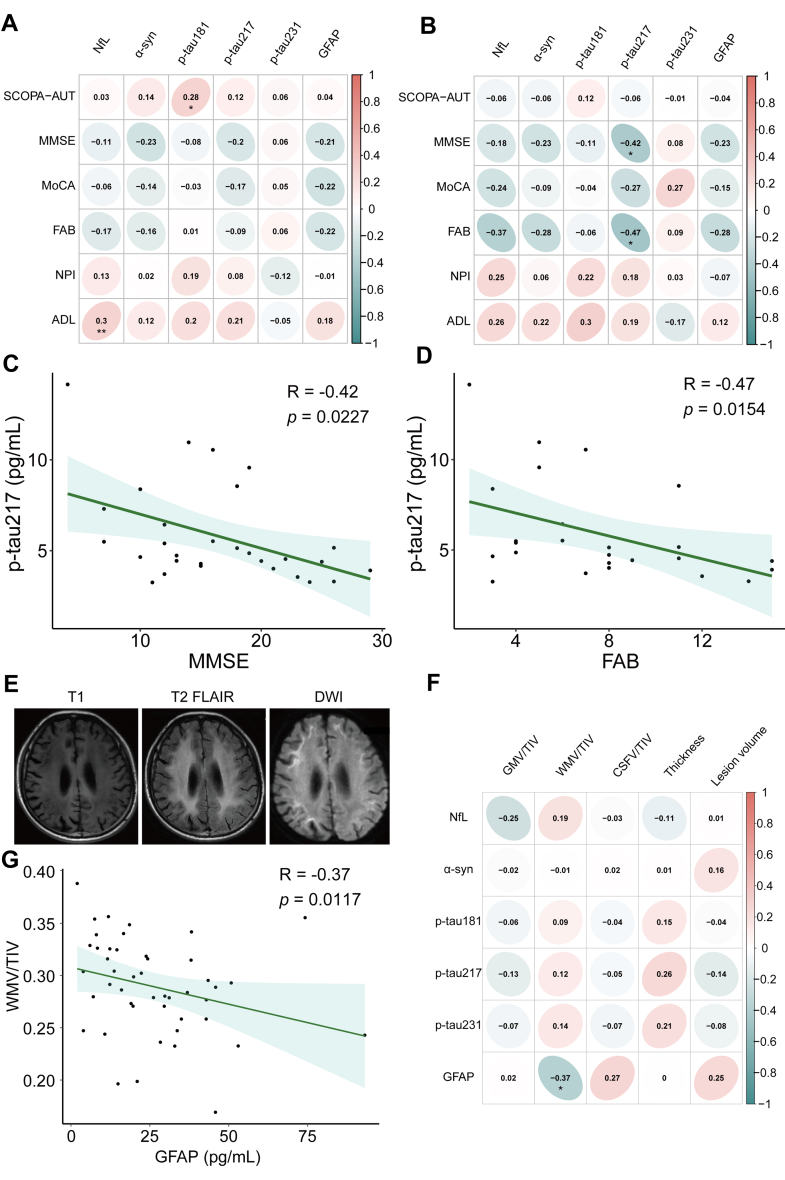
**Correlation between six plasma biomarkers and neuropsychological scores and neuroimaging measures in cohort 1.** (A) A significant positive correlation was observed between p-tau181 and SCOPA-AUT scores, as well as between NfL and ADL in NIID (n = 87). (B) MMSE and FAB showed a significantly negative correlation with p-tau217 in the dementia-dominant type of NIID (n = 34). (C, D) The monotonic correlation between MMSE (C) or FAB (D) and p-tau217. (E) The typical images of NIID. (F) The Spearman correlation between five MRI features and six plasma biomarkers, and plasma GFAP was significantly correlated with WMV, indicating that GFAP reflected the WMV atrophy in NIID (n = 51). (G) The monotonic correlation between plasma GFAP and WMV. The Spearman correlation analysis was used to evaluate the relevance. NIID, neuronal intranuclear inclusion disease; NfL, neurofilament light chain; α-syn, α-synuclein; p-tau, phosphorylated tau protein; GFAP, glial fibrillary acidic protein; SCOPA-AUT, Scales for Outcomes in Parkinson's Disease-Autonomic Questionnaire; MMSE, Mini-Mental State Examination; MoCA, Montreal Cognitive Assessment; FAB, Frontal Assessment Battery; NPI, Neuropsychiatric Inventory; ADL, Activity of Daily Living Scale. MRI, magnetic resonance imaging; FLAIR, fluid-attenuated inversion-recovery sequence; DWI, diffusion-weighted imaging; GMV, gray matter volume; WMV, white matter volume; CSFV, cerebrospinal fluid volume; TIV, total intracranial volume (∗p < 0.05, ∗∗p < 0.01).

We further analysed the correlation between the biomarkers and six psychological scores: SCOPA-AUT, MMSE, MoCA, FAB, NPI, and ADL. Overall, a statistically significant correlation between SCOPA-AUT and p-tau181 was observed in the NIID group, and NfL levels were positively correlated with ADL scores (Fig. 2A). We then calculated the correlation coefficients in the dementia-dominant and movement disorder-dominant subtypes, which are the two main subtypes of NIID. Interestingly, we found that p-tau217 showed a significant negative correlation with MMSE and FAB in dementia-dominant subtype (Fig. 2B), and the spearman correlation was further revealed in Fig. 2C and D. No correlation was observed for the movement disorder-dominant subtype (Figure S7).

### Association between plasma biomarkers and MRI features

To determine whether the altered plasma biomarkers were significantly correlated with MRI measures, we analysed the association between six neuroimaging measures (GMV, WMV, CSFV, cortical thickness, and lesion volume) and plasma biomarkers in patients with NIID. The typical MRI features are shown in Fig. 2E. Interestingly, GFAP levels were negatively correlated with the WMV, demonstrating that GFAP levels could reflect the severity of WMV atrophy in patients with NIID (Fig. 2F and G).

### Tau deposition in NIID

**Fig. 3:**
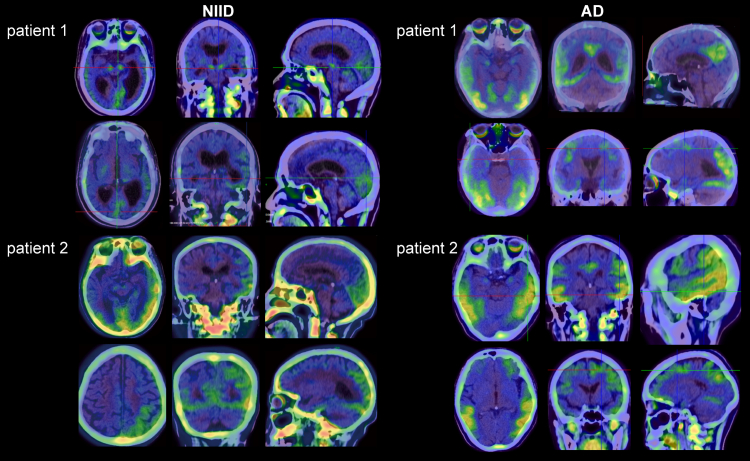
**Representative tau-PET imaging of patients with NIID and AD in cohort 1.** Pronounced tau tracer uptake in the substantia nigra and red nucleus of the midbrain region, the basal ganglia and the cerebral cortex in NIID (n = 10), whereas in patients with AD (n = 10), tau deposition is predominantly observed in the cerebral cortex.

Among patients with NIID, eight underwent Aβ PET/CT imaging, ten received tau PET/CT, and three were assessed with FDG PET/CT. Our findings revealed no detectable Aβ protein deposition in the cerebral cortex of patients with NIID (Figure S8). FDG-PET demonstrated reduced cerebral cortical metabolism in three patients, with bilateral hypometabolism in the caudate nuclei in two patients (Figure S9). Eight of the ten patients with NIID showed tau tracer uptake predominantly in the substantia nigra and red nucleus of the midbrain region, the basal ganglia and the cerebral cortex (Fig. 3). Specifically, two of these individuals with severe cognitive impairment exhibited elevated tau tracer uptake in the cortex (patients 1 and 2). Seven exhibited elevated tau tracer uptake in the substantia nigra and red nucleus of the midbrain region (patients 1, 3, 4, 5, 6, 7, and 8) (Figures S10, S11). Additionally, six patients had increased tau uptake signals in the basal ganglia (patients 1, 3, 4, 5, 7, and 8). In patients with AD, tau deposition is predominantly observed in the cerebral cortex (Fig. 3, Figure S10). To further validate tau deposition in NIID, voxel-wise analysis was performed. Notably, abnormal tau deposition on the occipital lobe and temporal pole was observed, whereas uptake in the substantia nigra, red nucleus, and basal ganglia failed to reach statistical significance (Figure S12). The clinical information of patients with NIID who completed tau-PET is presented in Table S5.

### P-tau and GFAP levels were significantly elevated in preNIID

**Fig. 4:**
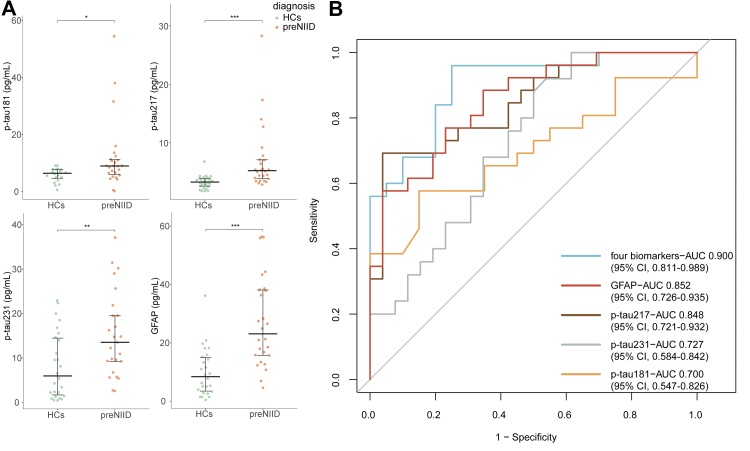
**Discriminating power of p-tau181, p-tau217, p-tau231 and GFAP levels between preNIID and HCs in cohort 2.** (A) The plasma p-tau181, p-tau217, p-tau231, and GFAP levels were significantly upregulated in the group of preNIID (n = 26) comparing to HCs (n = 26). (B) GFAP showed better performance than p-tau181, p-tau217, and p-tau231 levels in differentiating preNIID from HCs (AUC 0.852, 95% CI 0.726–0.935). A combination of these four biomarkers (GFAP, p-tau217, p-tau231, p-tau181) achieved an AUC of 0.900 (95% CI 0.811–0.989) in distinguishing preNIID from HCs. Mann–Whitney U test was used for comparison. Area under the curve (AUC) and confidence intervals were shown in figure. preNIID, presymptomatic NIID; HCs, healthy controls; p-tau, phosphorylated tau protein; GFAP, glial fibrillary acidic protein; AUC, the area under curve (∗p < 0.05, ∗∗p < 0.01, ∗∗∗p < 0.001).

To determine whether the values of these plasma biomarkers also significantly changed within individuals in the preNIID stage, we measured these eight indicators in 26 individuals with preNIID and 26 HCs in cohort 2. Among them, nine preNIID individuals completed the MRI scan, and no white matter hyperintensities or brain atrophy were identified (Figure S13). On comparing of preNIID and sex- and age-matched HCs, we found that p-tau181, p-tau217, p-tau231, and GFAP levels were significantly higher in preNIID than HCs (Fig. 4A), while Aβ40, Aβ40, NfL, and α-syn were unchanged in preNIID (Figure S14). We further analysed the difference of p-tau/Aβ42 and Aβ42/40 ratio between HCs and preNIID individuals. While no statistically significant difference was observed (Figure S15). ROC curve analysis was performed for differential diagnosis, and we found that the AUC of GFAP (0.852, 95% CI, 0.726–0.935) was the highest among the four significantly altered biomarkers under a cut-off value of 15.23 pg/mL, followed by p-tau217 (AUC 0.848, 95% CI, 0.721–0.932) at a cut-off value of 4.34 pg/mL, p-tau231 (AUC 0.727, 95% CI, 0.584–0.842) at a cut-off value of 2.40 pg/mL, and p-tau181 (AUC 0.700, 95% CI, 0.547–0.826) at a cut-off value of 8.05 pg/mL (Fig. 4B). The combination of GFAP, p-tau217, p-tau231, and p-tau181 achieved an AUC of 0.900 (95% CI, 0.811–0.989) in distinguishing individuals with preNIID from HCs, indicating that p-tau and GFAP alterations are early indicators of NIID pathogenesis.

## Discussion

In our study, eight plasma biomarkers were detected in two cohorts: cohort 1 with 87 NIID cases and 147 AD cases, and cohort 2 with 26 preNIID cases. Alterations in p-tau species were prominent in patients with NIID, especially p-tau217. Moreover, p-tau and GFAP levels were significantly upregulated in both the NIID and preNIID groups and correlated with disease severity. A pronounced tau deposition was noted in NIID. This study expanded the biomarker spectrum of NIID, and demonstrated an early alteration of plasma p-tau and GFAP in the preclinical stage of NIID.

P-tau alteration, especially p-tau217, is a promising biomarker for detecting AD pathology due to its close association with both brain Aβ and early tau pathology.^17^^,^^18^ Previous studies have also demonstrated that p-tau217 outperforms p-tau181 in distinguishing AD from other neurodegenerative diseases, including Parkinson's disease (PD), progressive supranuclear palsy (PSP), frontotemporal dementia and vascular dementia.^19^ While the p-tau alteration in NIID has rarely been reported. Our study found that the plasma p-tau species, especially p-tau217 in patients with NIID were significantly rising. However, the plasma p-tau species were difficult in distinguishing NIID from AD. Changes in plasma p-tau levels have been observed in several neurological disorders other than AD, including corticobasal syndrome,^20^ amyotrophic lateral sclerosis,^21^ and other conditions such as epilepsy,^22^ and traumatic encephalopathy syndrome.^23^ Notably, increased plasma p-tau levels in these neurodegenerative diseases is often associated with Aβ deposition in the brain.^20^^,^^21^^,^^23^ However, no Aβ deposition was detected on PET/CT imaging in patients with NIID, which is consistent with previous study.^11^ It has also been reported that Aβ staining was negative in brain regions of patients with NIID,^24^ and CSF examination for Aβ was negative in NIID cases.^24^^,^^25^ Furthermore, the AT(N) classification of patients with NIID demonstrated that up to 75% of cases were classified as A-T+.^11^ These studies provide new evidence that p-tau alterations, especially p-tau217, in NIID may be driven by a novel mechanism independent of amyloid pathology in the pathogenesis of NIID. A recent study identified a (GGGAGA)n repeat expansion in the intron of the *CASP8* gene as a novel genetic risk factor for AD. This repeat expansion leads to the production of poly-glycine-arginine (polyGR) protein aggregates, which positively correlate with AT8 immunoreactivity in the AD hippocampus. Overexpression of polyGR in SH-SY5Y cell models markedly increased p-tau levels, suggesting that polyGR may promote tau/p-tau aggregation.^26^ Given that the GGC repeat expansion in the NOTCH2NLC gene-known as the pathogenic gene of NIID-can be translated into poly-glycine (polyG) protein,^27^, ^28^, ^29^ we hypothesise that polyG may induce tau deposition in NIID through a similar mechanism, thus warranting further exploration.

Our tau PET/CT scan further confirmed tau deposition in the brain of patients with NIID. The pathological features of NIID and AD are distinct, and NIID is currently considered as a polyG disease.^27^, ^28^, ^29^ Our study provides the *in vivo* evidence of pronounced tau deposition in the cortex in NIID. Tau tracer uptake in the midbrain regions, including the substantia nigra and red nucleus, was not statistically significant. This pattern aligns with the characteristic of 18F-MK6240, which exhibits physiological uptake in these specific areas.^30^ In contrast, AD-related tau pathology, as visualised through 18F-MK6240 tau-PET, predominantly follows a characteristic spatiotemporal progression pattern involving the transentorhinal cortex, entorhinal cortex, hippocampus, temporal neocortex, association cortices, and primary sensory areas^31^; this pattern is distinct from that observed in NIID. A prior neuropathological study demonstrated absent tau immunoreactivity in NIID brain specimens, including cortical, hippocampal, striatal, and thalamic regions.^24^ The limited sample size may explain the observed tau-negative pathology in the previous study, highlighting the need for further brain pathology examinations. Moreover, 18F-MK6240 used in this study was believed to be less sensitive to 4R-tauopathy, such as PSP,^30^ indicating that further exploration of the tau splicing isoforms (3R tau and 4R tau) in NIID is warranted. We hypothesise that tauopathy may arise secondary to polyG pathology, a hallmark of NIID. Mechanistically, polyG interacted with FUS, disrupting stress granule formation in cytoplasm,^32^ and it tends to aggregate with the increase of GGC repeat units and displays liquid–liquid phase separation (LLPS) properties.^28^ A previous study has demonstrated that LLPS induces pathogenic tau conformations.^33^ Therefore, the pronounced tau deposition observed in NIID could arise secondarily from polyG pathology through LLPS or other novel mechanisms.

GFAP is expressed in mature astrocytes, and acts as a well-established marker of astrocyte injury and activation in several neurological diseases. It has been extensively explored in the context of AD, where plasma GFAP levels increase in the preclinical phase and may serve as an early biomarker for dementia prediction.^34^, ^35^, ^36^, ^37^, ^38^, ^39^ Notably, GFAP alterations occur before changes in plasma p-tau or NfL in AD^37^ and it may link Aβ with initial tau pathology in AD, making it a valuable marker in AD progression.^35^ Previous studies on a Chinese cohort of 30 patients with NIID revealed that plasma GFAP levels were notably higher in the NIID group than in HCs.^40^ In this study, we observed that plasma GFAP distinguished NIID from HCs and increased before the onset of clinical symptoms, thereby highlighting its potential as a diagnostic and predictive biomarker for NIID. Previous autopsy studies have reported that inclusion-bearing astrocytes are more affected than neurons in adult-onset NIID,^13^^,^^14^ which provides pathological evidence supporting the significant increase in plasma GFAP levels in the NIID group. Additionally, brain atrophy is commonly observed in NIID,^4^^,^^41^ and we found a strong correlation between GFAP levels and WMV, indicating that plasma GFAP levels correlate with disease severity of NIID.

NfL is a validated biomarker of neuroaxonal injury and has been extensively explored in various neurodegenerative diseases.^42^^,^^43^ Our study further highlights its clinical value in diagnosing NIID.^44^, ^45^, ^46^, ^47^, ^48^ Our study found that both GFAP and NfL are useful biomarkers for distinguishing NIID and AD. As reported previously, 66.8% of patients with NIID presented with paroxysmal symptoms,^4^ and NfL levels were notably higher in acute-onset NIID, whereas no statistical difference was observed for GFAP levels.^40^ Therefore, the significant upregulation of NfL in NIID may be attributed to the presence of paroxysmal symptoms in these patients. We also found that α-syn levels were significantly higher in the NIID group, thereby providing a biomarker for revealing disease pathology. A prominent correlation between abnormal GGC repeats in NOTCH2NLC and clinically diagnosed PD has been reported.^49^, ^50^, ^51^, ^52^, ^53^ Since patients carrying NOTCH2NLC mutations were clinically diagnosed with PD according to Movement Disorder Society clinical diagnostic criteria or UK Brain Bank criteria, these patients could be defined as NIID presenting with parkinsonism. Additionally, patients with PD carrying NOTCH2NLC mutation also exhibited a graded and asymmetrical reduction in DAT binding in the putamen. Dopaminergic neuron degeneration in the substantia nigra was noted in *NOTCH2NLC*-(GGC)_98_ transgenic mice in an age-dependent manner^54^; and the NOTCH2NLC GGC intermediate repeat with serine could induce early Parkinson's disease-like phenotypes in mice,^55^ further indicating a pathological overlap between PD and NIID.

This study has some limitations. First, the limited sample size of the preNIID group may have influenced the plasma biomarker spectrum. Second, the absence of a significant difference in plasma Aβ levels between AD and HC groups partially limits the conclusion regarding the lack of amyloid pathology in NIID, despite the supporting negative Aβ-PET results. Third, the mechanisms underlying the alterations of specific biomarkers require further investigation.

In conclusion, our study found elevated levels of plasma p-tau species, α-syn, NfL, and GFAP in NIID. The multiple biomarker changes indicate the complexity of NIID pathology. Elevated plasma p-tau species and GFAP levels in the preclinical stage of NIID suggest their potential as predictive markers for monitoring disease progression. The comparable p-tau levels, especially p-tau217, between NIID and AD made differentiating NIID from AD difficult, questioning p-tau217's specificity as an AD-exclusive biomarker. Tau deposition detected by PET/CT provides evidence for underlying tau pathology in NIID. Further exploration into this novel mechanism of tau deposition in NIID pathogenesis is warranted.

## Contributors

The enrolment of patients was supported by Sizhe Zhang, Yan Zeng, Lu Zhou, Yun Tian, Bin Jiao, Qiying Sun, Ling Weng, Juan Du, Qian Xu, Yang Yang, Mengqi Zhang, Qiuming Zeng, Liangjuan Fang, Hongyu Long, Yuanyuan Xie, Si Chen, Li Feng, Qing Huang, Lili Long, Yafang Zhou, Fang Yi, Yacen Hu, Qiong Liu, Yongcheng Pan, Lin Zhou, Jifeng Guo, Junling Wang, Hong Jiang, Hongwei Xu, Beisha Tang, and Lu Shen. Sizhe Zhang and Yun Tian drafted the manuscript. The genetic diagnosis was supported by Ranhui Duan and Qiao Xiao. The plasma biomarkers detection and analysis were supported by Sizhe Zhang, Bin Jiao, Ziyu Ouyang, Yunni Li and Yun Tian. The data of MRI was analysed by Xiaoyu Chen and Weiwei Zhang. The PET/CT data processing was supported by Shuo Hu and Yulai Li. Qiong Liu, Yongcheng Pan, Bin Jiao, Beisha Tang, and Lu Shen made critical revisions of the manuscript. Lu Shen and Yun Tian supervised the completion of this study. Lu Shen and Yun Tian accessed and verified the underlying data. All authors read and approved the final version of the manuscript.

## Data sharing statement

All data and materials used in the analysis could be available from the correspondence authors for purposes of reproducing or extending the analysis.

## Declaration of interests

All authors declare no financial or non-financial competing interests.
