# Supplementary material for: Plasma p-tau species are elevated in presymptomatic and symptomatic neuronal intranuclear inclusion disease
Source: eBioMedicine. 2026 Jan 14;124:106127. doi: 10.1016/j.ebiom.2026.106127 (PMC12830138; doi:10.1016/j.ebiom.2026.106127)
Supplement: Supplementary Material [file mmc1.docx]

Supplementary Methods and results

[Section 1 Supplementary Methods 2](#_Toc216537790)

[Supplementary Table 1 Relevant diagnostic data for AD and NIID in cohort 1. 2](#_Toc216537791)

[Supplementary Table 2 Biomarker diluent ladder for standard curve building. 3](#_Toc216537792)

[Supplementary Figure 1 Fitted standard curves for each biomarker. 3](#_Toc216537793)

[Supplementary Table 3 LOD and LOQ results. 4](#_Toc216537794)

[Section 2 Supplementary Results 5](#_Toc216537795)

[Supplementary Figure 2 Plasma p-tau/Aβ42 ratio and Aβ42/40 ratio among HCs, NIID and AD in cohort 1. 5](#_Toc216537796)

[Supplementary Figure 3 Correlation between age at onset (AAO) and the six altered plasma biomarkers in cohort 1. 6](#_Toc216537797)

[Supplementary Figure 4 Correlation between disease duration and the six altered plasma biomarkers in cohort 1. 7](#_Toc216537798)

[Supplementary Figure 5 Correlation between GGC repeat length and the six altered plasma biomarkers in cohort 1. 8](#_Toc216537799)

[Supplementary Figure 6 Comparison of eight plasma biomarkers among patients with NIID in four different clinical phenotypes in cohort 1. 9](#_Toc216537800)

[Supplementary Figure 7 Correlation between six plasma biomarkers and neuropsychological scores in the movement disorder-dominant subtype in cohort 1. 10](#_Toc216537801)

[Supplementary Figure 8 Aβ-PET imaging of patients with NIID in cohort 1. 11](#_Toc216537802)

[Supplementary Figure 9 FDG-PET imaging of patients with NIID in cohort 1. 12](#_Toc216537803)

[Supplementary Figure 10 tau-PET and corresponding Aβ-PET imaging of patients with NIID and AD in cohort 1. 13](#_Toc216537804)

[Supplementary Figure 11 axial cuts of tau-PET imaging of patients with NIID in cohort 1. 15](#_Toc216537805)

[Supplementary Figure 12 Distribution of increased 18F-MK6240 uptake identified by voxel-wise analysis in patients with NIID relative to healthy controls (HCs). 16](#_Toc216537806)

[Supplementary Figure 13 T2-FLAIR sequence of nine preNIID individuals in cohort 2. 17](#_Toc216537807)

[Supplementary Figure 14 No significant difference was observed in plasma Aβ40, Aβ42, NfL, and α-syn levels between preNIID and HCs in cohort 2. 18](#_Toc216537808)

[Supplementary Figure 15 Comparison of p-tau/Aβ42 and Aβ42/40 ratios between HCs and preNIID individuals in cohort 2. 19](#_Toc216537809)

[Supplementary Table 4 The basic information of four phenotypes of NIID in cohort 1 20](#_Toc216537810)

[Supplementary Table 5 Clinical information of ten patients with NIID completing tau-PET scan 21](#_Toc216537811)

[Reference 23](#_Toc216537812)

# Section 1 Supplementary Methods

## Supplementary Table 1 Relevant diagnostic data for AD and NIID in cohort 1.

|  | | AD (n = 147) | NIID (n = 87) |
| --- | --- | --- | --- |
| Aβ-PET | | 32 positive | - |
| CSF (n = 115) | Aβ42 | 355.45 [253.92; 475.45] | - |
|  | Aβ42/40 | 0.06 [0.04; 0.08] | - |
|  | p-tau181 | 72.405 [35.63; 118.825] | - |
|  | t-tau | 331.99 [201.94; 552.53] | - |
| GGC repeats number | | - | 118 [99; 142] |

**Single Molecular Immunity Detection Methods (the AST-Sc-Lite) as described by Bin Jiao et al ^1^.**

The standards for Aβ1-40, Aβ1-42, p-tau181, p-tau217, p-tau231, NfL, GFAP and α-syn are diluted in a commercial plasma matrix with biomarkers removed, as shown in the **Supplementary Table 2** below. Each diluted sample was measured three times using the following steps on AST-Sc-Lite platform:

Step 1: Load 25 μL of sample into an incubation tube, and add 25 μL of Reagent 1 (containing 0.1 mg/mL magnetic beads coated with capture antibodies and protective reagents). The sample is then automatically mixed by the machine.

Step 2: After 6 min of incubation, 10 μL of Reagent 2 (containing detection antibodies labelled with single-molecule imaging fluorophores) is added, mixed, and incubated for 4 min at 40°C.

Step 3: The magnetic beads in the mixture are captured onto the surface of the channel in the flow cell using a permanent magnet. Unlabelled fluorophores are washed away by a gentle flow of wash buffer, and fluorescent images are captured with an integrated fluorescent microscope.

Step 4: Standard curves for each plasma biomarker are generated using custom fitting software (**Supplementary Figure 1**).

## Supplementary Table 2 Biomarker diluent ladder for standard curve building.

| Aβ40 | Aβ42 | p-Tau181 | p-Tau217 | p-Tau231 | NFL | GFAP | α-syn |
| --- | --- | --- | --- | --- | --- | --- | --- |
| pg/mL | | | | | | | |
| 0 | 0 | 0 | 0 | 0 | 0 | 0 | 0 |
| 5.65 | 5.08 | 2.82 | 0.56 | 0.28 | 5.08 | 5.08 | 16.94 |
| 16.94 | 15.24 | 8.47 | 1.69 | 0.85 | 15.24 | 15.24 | 50.81 |
| 50.81 | 45.72 | 25.4 | 5.08 | 2.54 | 45.72 | 45.72 | 152.42 |
| 152.42 | 137.17 | 76.21 | 15.24 | 7.62 | 137.17 | 137.17 | 457.25 |
| 457.25 | 411.52 | 228.62 | 45.72 | 22.86 | 411.52 | 411.52 | 1371.74 |
| 1371.74 | 1234.57 | 685.87 | 137.17 | 68.59 | 1234.57 | 1234.57 | 4115.23 |
| 4115.23 | 3703.7 | 2057.61 | 411.52 | 205.76 | 3703.7 | 3703.7 | 12345.7 |
| 12345.7 | 11111.1 | 6172.84 | 1234.57 | 617.28 | 11111.1 | 11111.1 | 37037 |
| 37037 | 33333.3 | 18518.5 | 3703.7 | 1851.85 | 33333.3 | 33333.3 | 111111 |
| 111111 | 100000 | 55555.6 | 11111.1 | 5555 | 100000 | 100000 | 333333 |

## Supplementary Figure 1 Fitted standard curves for each biomarker.

**
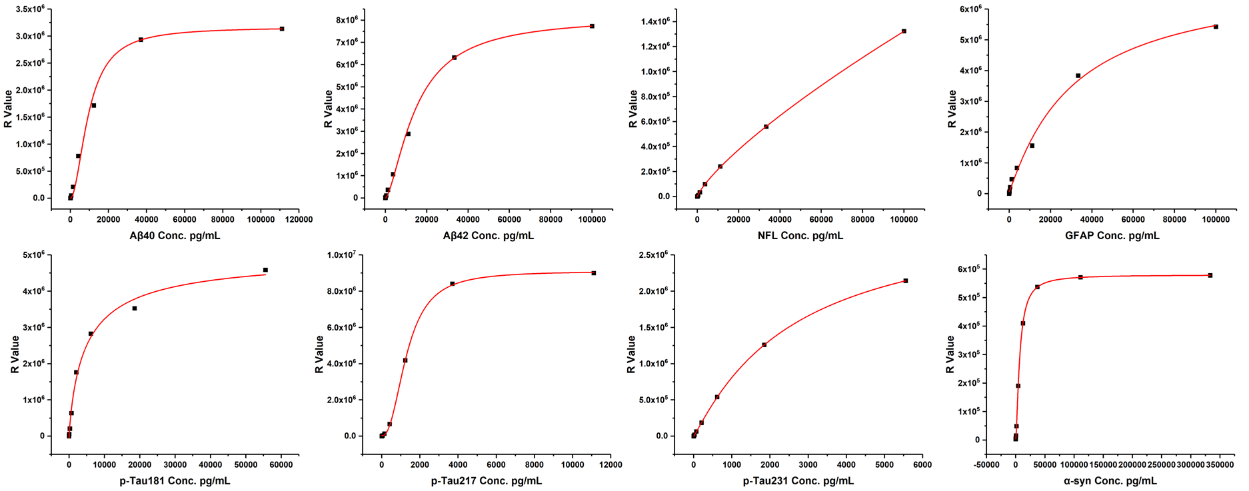
**

The LOD and LOQ results for 10 min incubation are presented in the **Supplementary Table 3** below. The LOD was determined by 2SD*_blank_*/S, where SD*_blank_* is the standard deviation of 20 measurements of the dilution buffer for each biomarker, and S is the slope of the linear regression formula near the blank. To determine the LOQ, a series of samples with varying concentrations of each biomarker were prepared and measured five times each. The concentrations at which the coefficient of variation (CV%) was approximately 20% was identified as the LOQ.

## Supplementary Table 3 LOD and LOQ results.

|  | **LOD** | **LOQ** |
| --- | --- | --- |
| **P-tau181** | 0.12 pg/mL | 0.319 pg/mL |
| **P-tau217** | 0.096 pg/mL | 0.252 pg/mL |
| **P-tau231** | 0.53 pg/mL | 0.87 pg/mL |
| **GFAP** | 0.61 pg/mL | 1.30 pg/mL |
| **NfL** | 0.95 pg/mL | 1.68 pg/mL |
| **α-syn** | 15.8 pg/mL | 53.3 pg/mL |

# Section 2 Supplementary Results

## Supplementary Figure 2 Plasma p-tau/Aβ42 ratio and Aβ42/40 ratio among HCs, NIID, and AD in cohort 1.

The p-tau/Aβ42 ratio showed statistically significant differences between NIID (n = 87) and HCs (n = 110), as well as between AD (n = 147) and HCs (n = 110). However, no difference was observed in the p-tau/Aβ42 ratio between NIID and AD. No differences were found in the Aβ42/40 ratio across these three groups. Benjamini & Hochberg adjusted Kruskal-Wallis H test was used for pairwise comparisons.


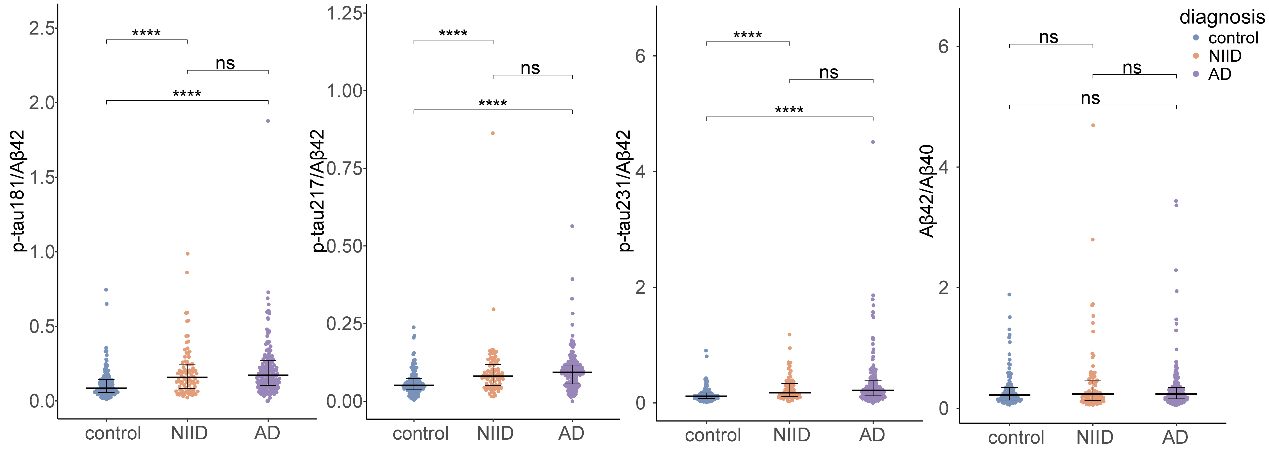


## Supplementary Figure 3 Correlation between age at onset (AAO) and the six altered plasma biomarkers in cohort 1.

GFAP exhibited a positive correlation with disease AAO (n = 87). The Spearman correlation analysis was used to evaluate the relevance.


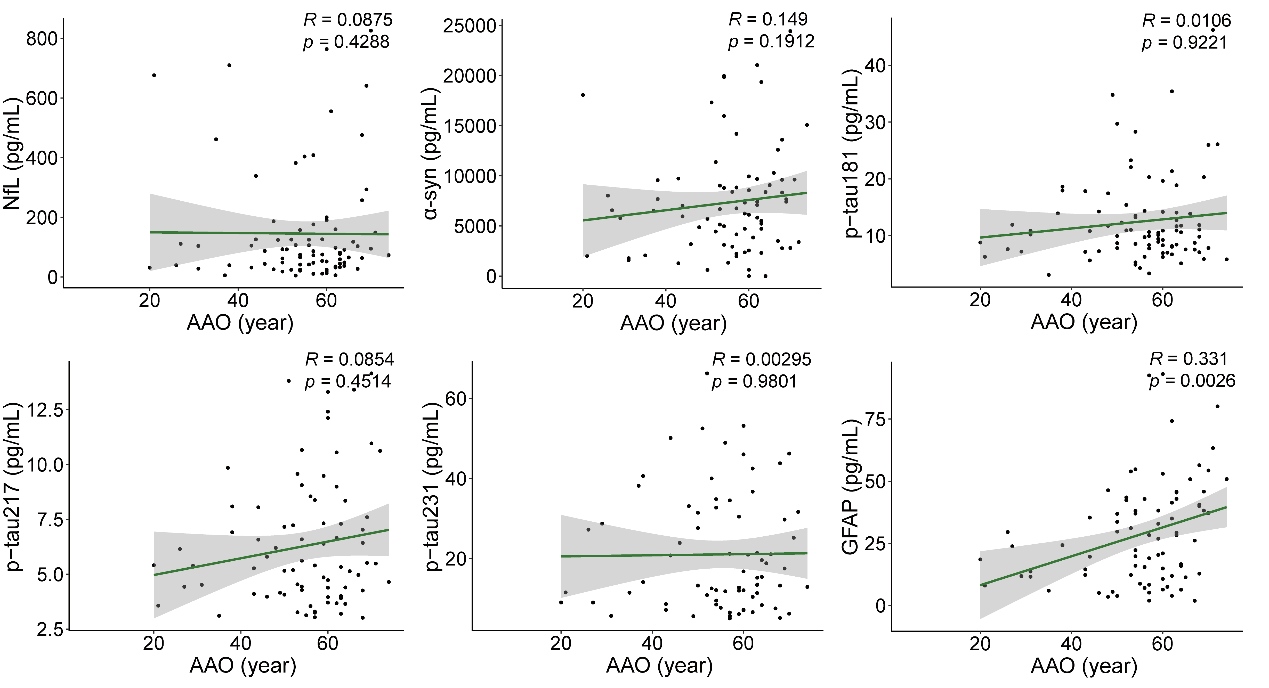


## Supplementary Figure 4 Correlation between disease duration and the six altered plasma biomarkers in cohort 1.

No significant correlations were observed (n = 87). The Spearman correlation analysis was used to evaluate the relevance.


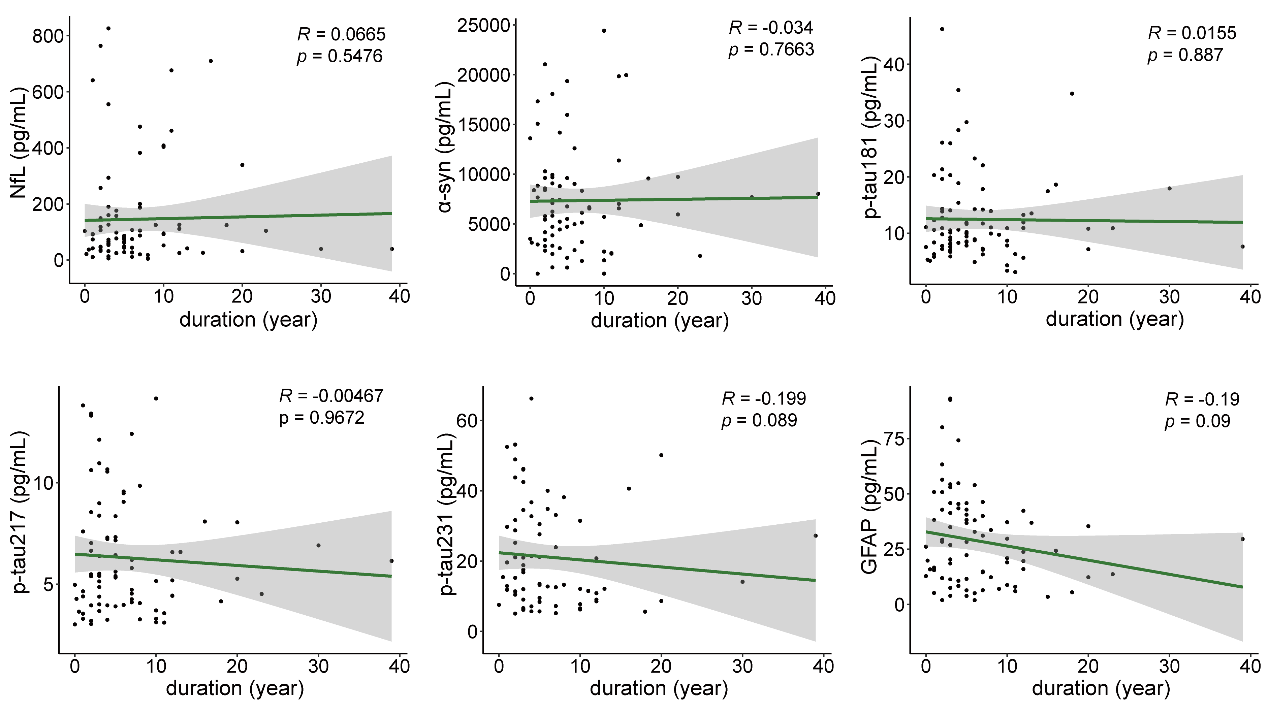


## Supplementary Figure 5 Correlation between GGC repeat length and the six altered plasma biomarkers in cohort 1.

No significant correlation was observed (n = 87). The Spearman correlation analysis was used to evaluate the relevance.


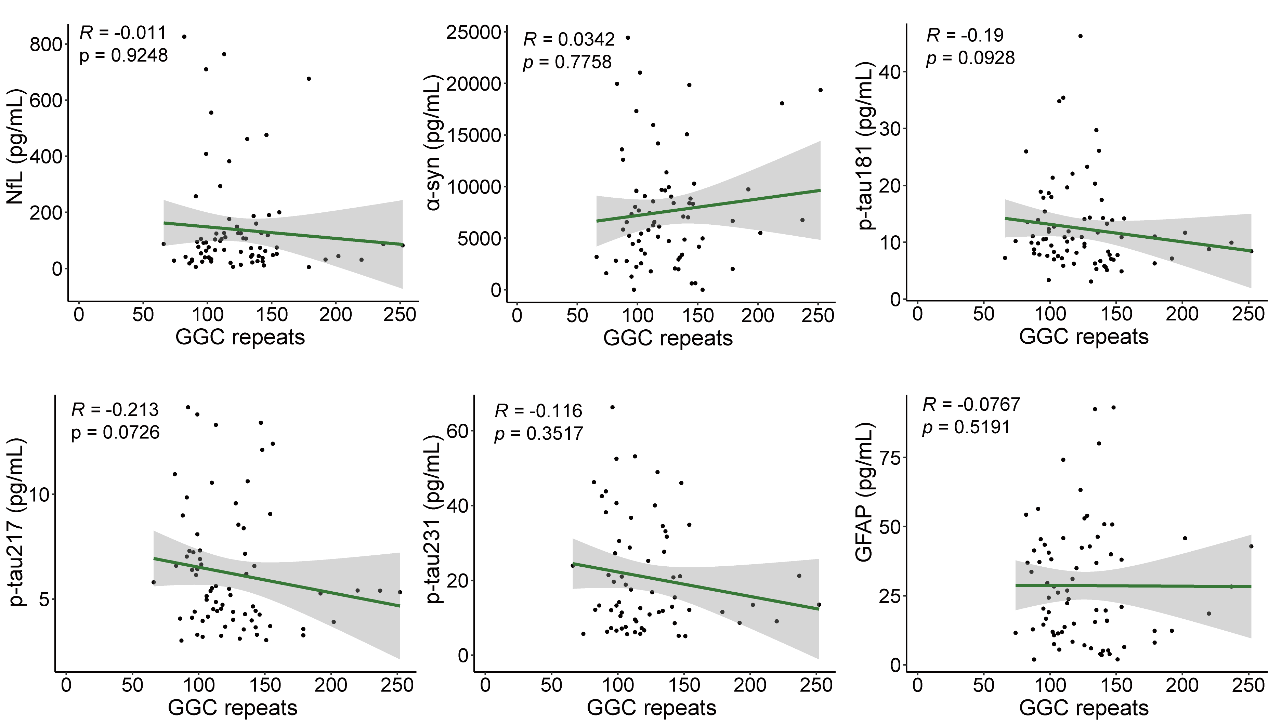


## Supplementary Figure 6 Comparison of eight plasma biomarkers among patients with NIID in four different clinical phenotypes in cohort 1.

No significant difference was observed including muscle weakness-dominant subtype (n = 9), dementia-dominant subtype (n = 34), movement disorder-dominant type (n = 30), and paroxysmal symptom-dominant type (n = 14). Statistics were acquired through Benjamini & Hochberg adjustment method with Kruskal-Wallis H test for pairwise comparisons.

**
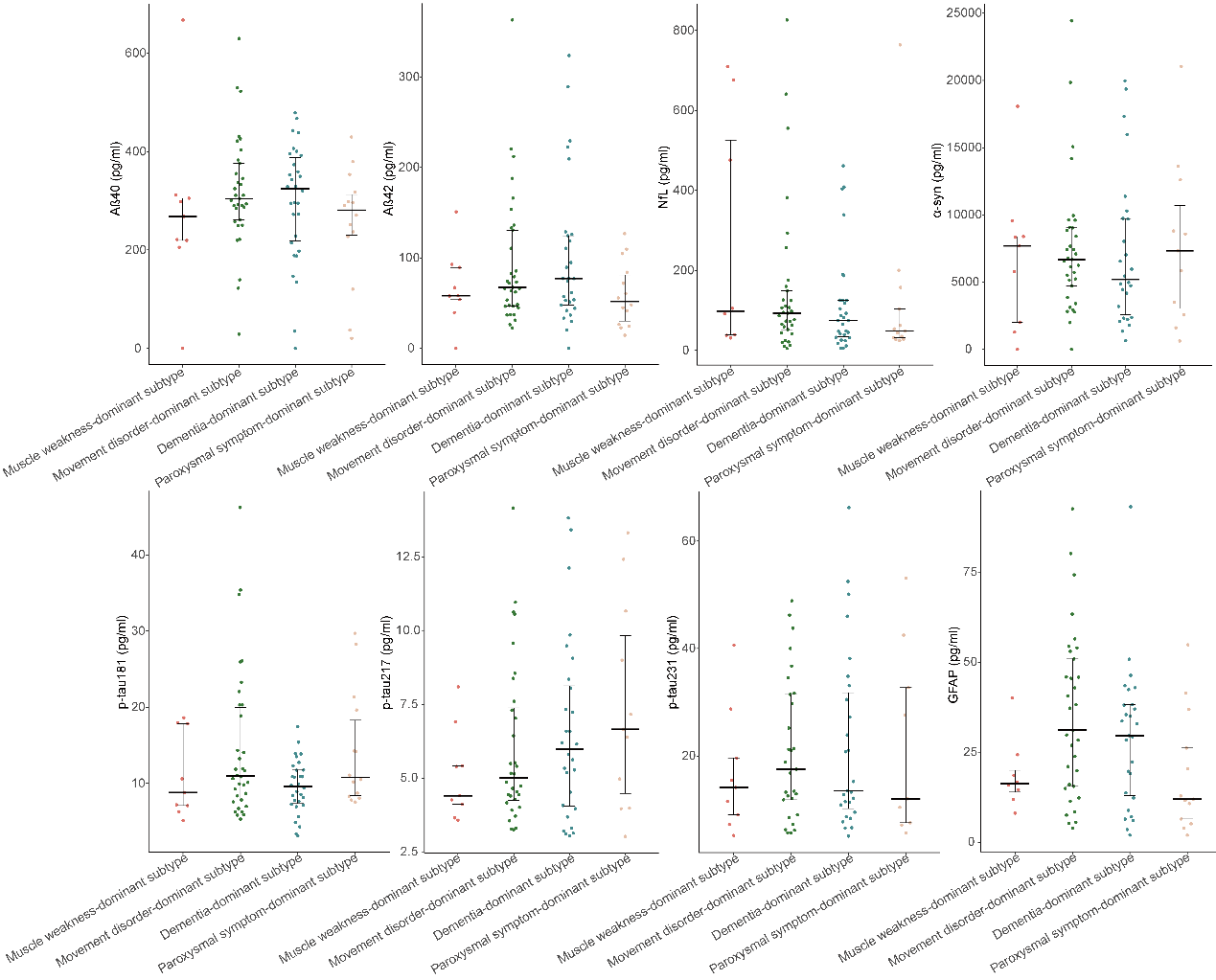
**

## Supplementary Figure 7 Correlation between six plasma biomarkers and neuropsychological scores in the movement disorder-dominant subtype in cohort 1.

No statistically significant correlation was observed in the movement disorder dominant type of the patients with NIID (n = 30). The Spearman correlation analysis was used to evaluate the relevance.

**
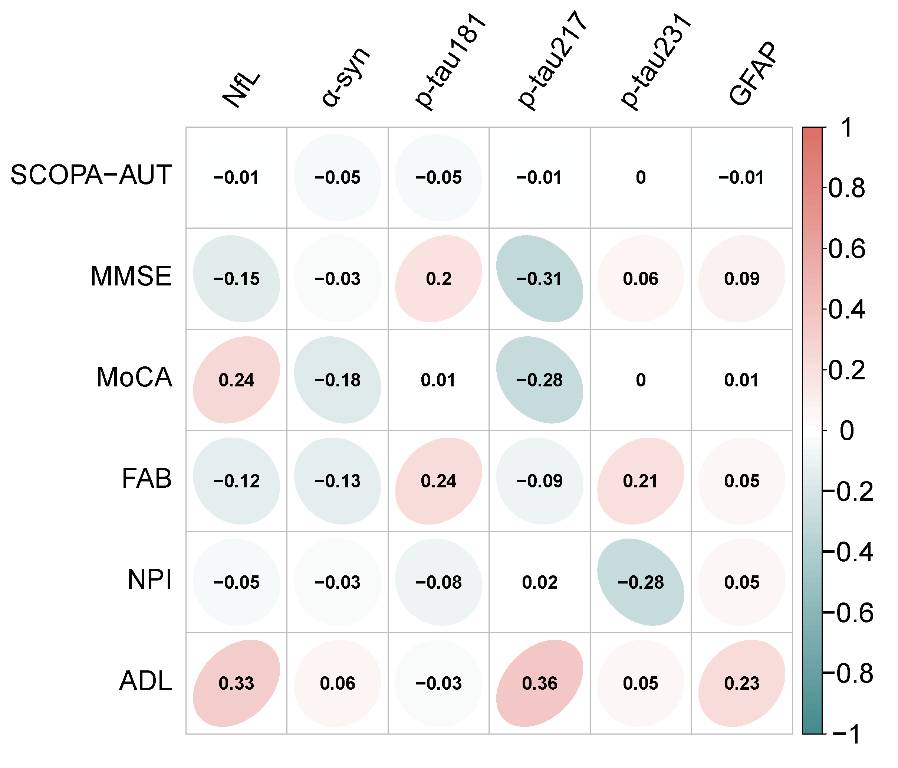
**

## Supplementary Figure 8 Aβ-PET imaging of patients with NIID in cohort 1.

No Aβ deposition was detected across eight NIID cases (n = 8), different from patients with AD (n = 10).


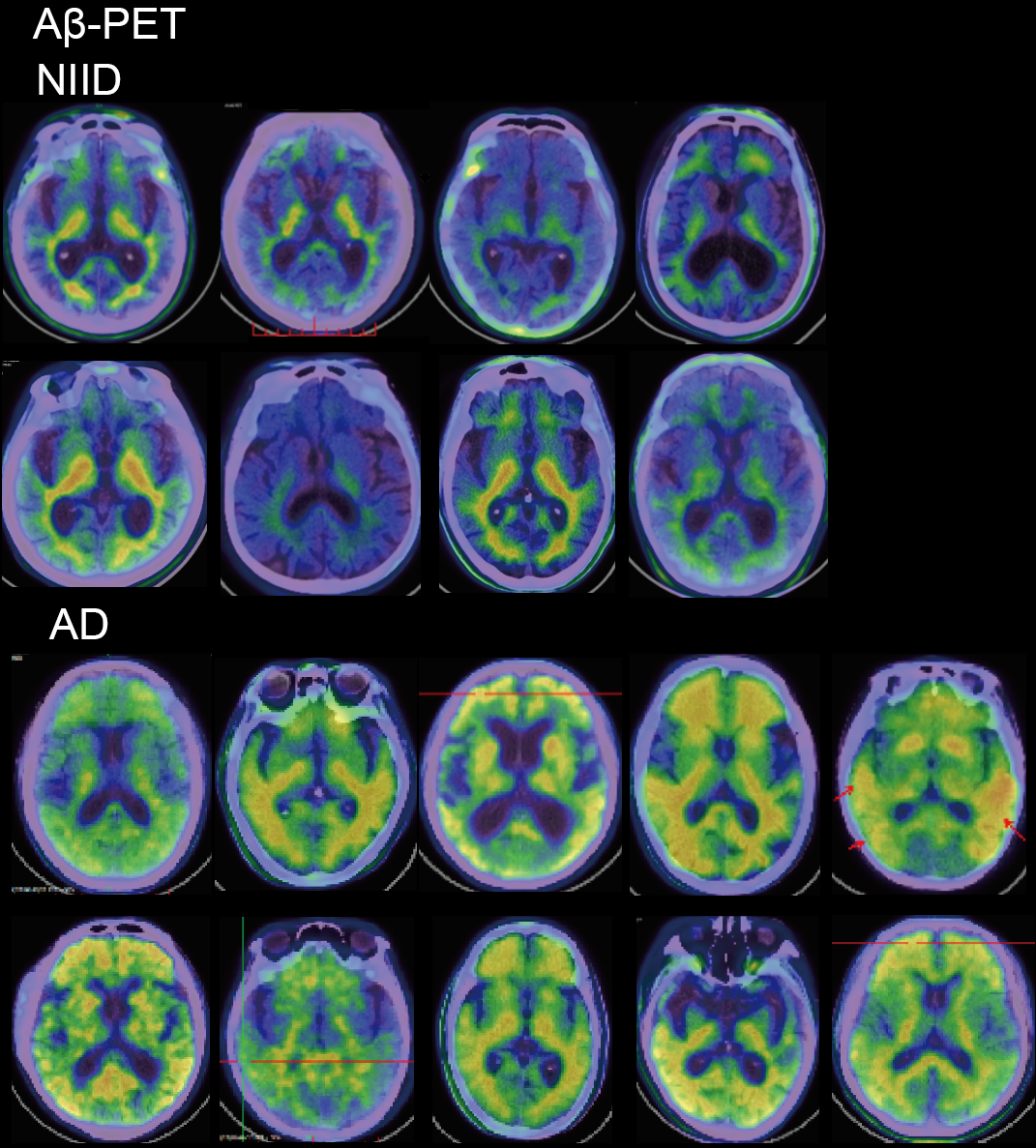


## Supplementary Figure 9 FDG-PET imaging of patients with NIID in cohort 1.

Reduced cerebral cortical metabolism was observed through FDG-PET in three patients (n = 3), with bilateral hypometabolism in the caudate nuclei in two patients.

## Supplementary Figure 10 Tau-PET and corresponding Aβ-PET imaging of patients with NIID and AD in cohort 1.

In NIID, pronounced tau deposition was localised to the frontal, parietal, occipital, and temporal lobes in patient 1 and the parietal, occipital, and temporal lobes in patient 2 (The tau-PET images of patient 1 and 2 were shown in **Figure 3** as representatives). Elevated tau deposition was observed in the basal ganglia (patients 1, 3, 4, 5, 7, and 8). In patients 1, 3, 4, 5, 6, 7, and 8, a putative tau signal was found in the substantia nigra and red nucleus of the midbrain. Two patients with intact cognitive function exhibited no abnormal tau deposition (patients 9 and 10). The Aβ-PET were negative in the eight patients with NIID. In AD, pronounced Aβ deposition was observed in patients with AD (n = 10), and the tau deposition was mainly observed in cortex (n = 10).

**
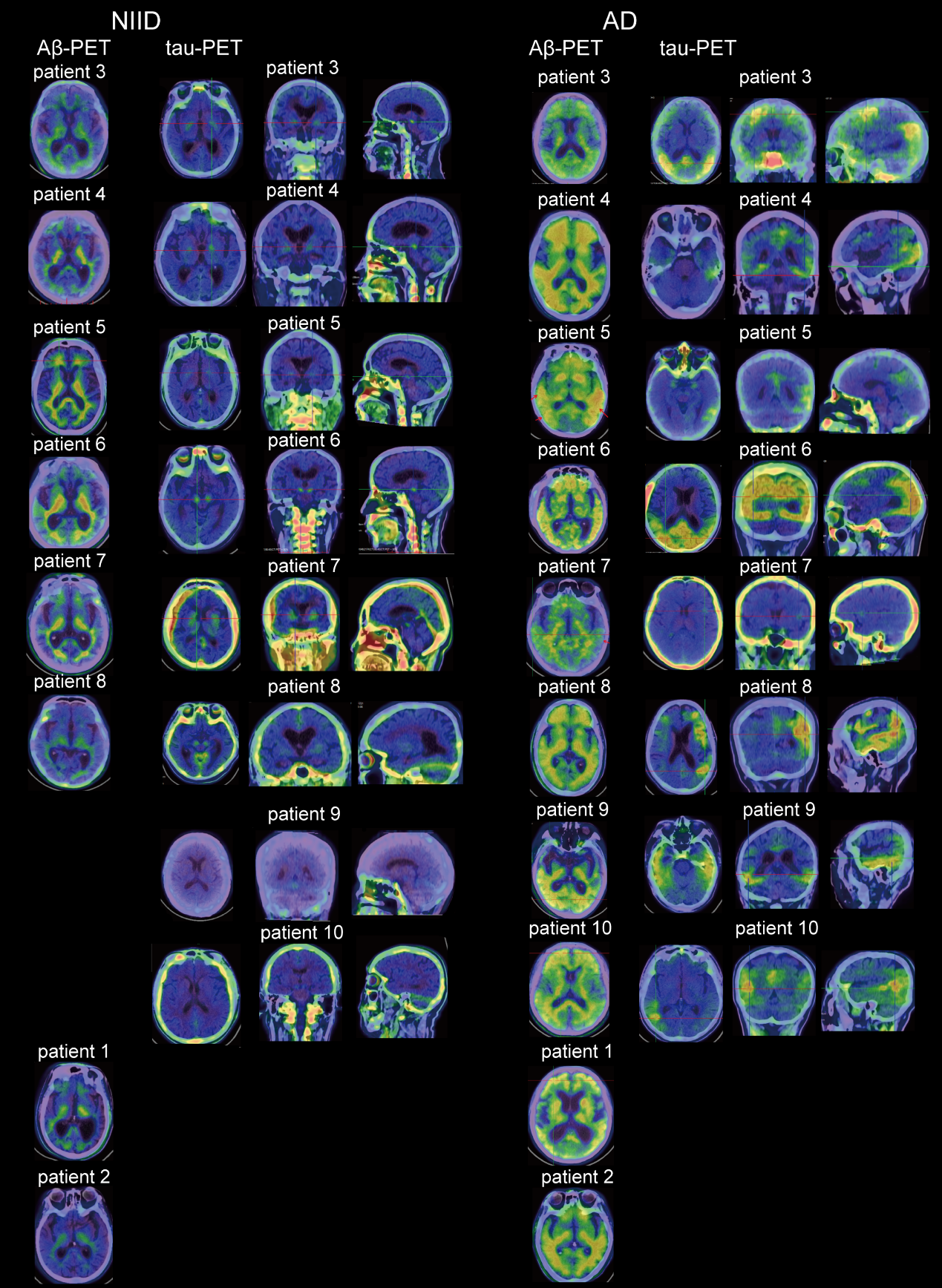
**

## Supplementary Figure 11 Axial cuts of tau-PET imaging of patients with NIID in cohort 1.

**
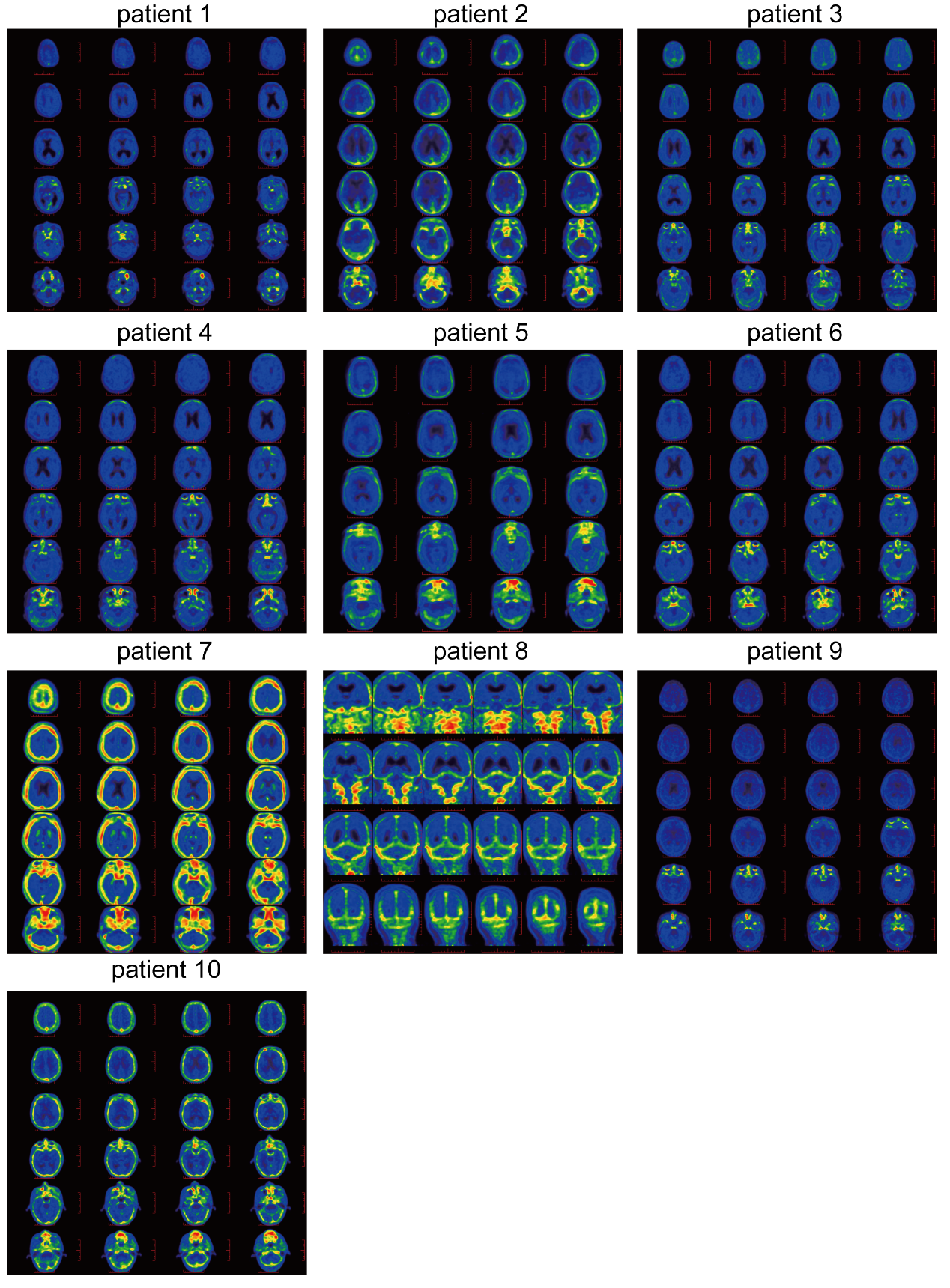
**

## Supplementary Figure 12 Distribution of increased 18F-MK6240 uptake identified by voxel-wise analysis in patients with NIID relative to healthy controls (HCs).

Group differences were assessed with a two-tailed unpaired t-test.

**
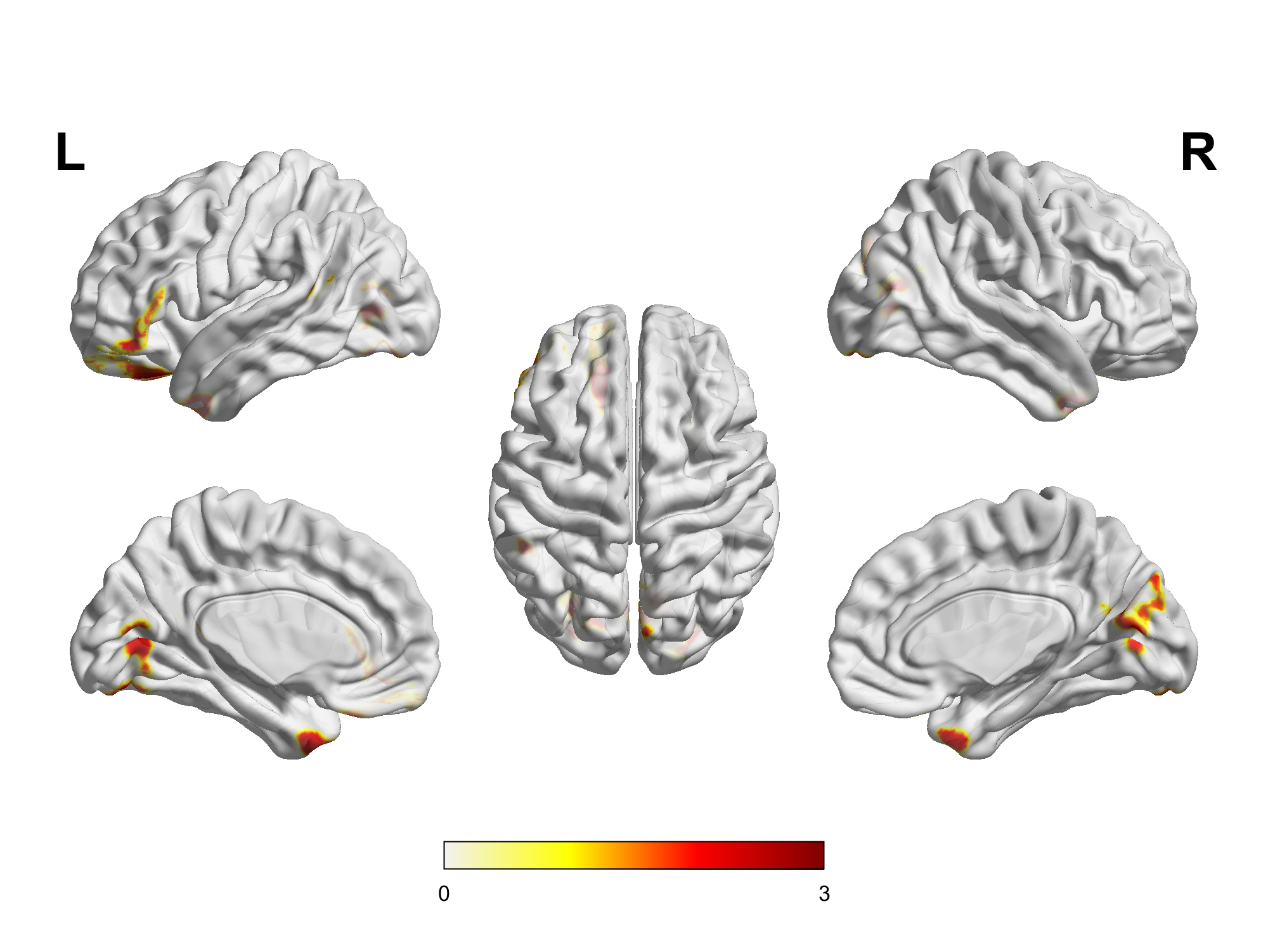
**

## Supplementary Figure 13 T2-FLAIR sequence of nine preNIID individuals in cohort 2.

No white matter hyperintensities or brain atrophy were identified (n = 9).

**
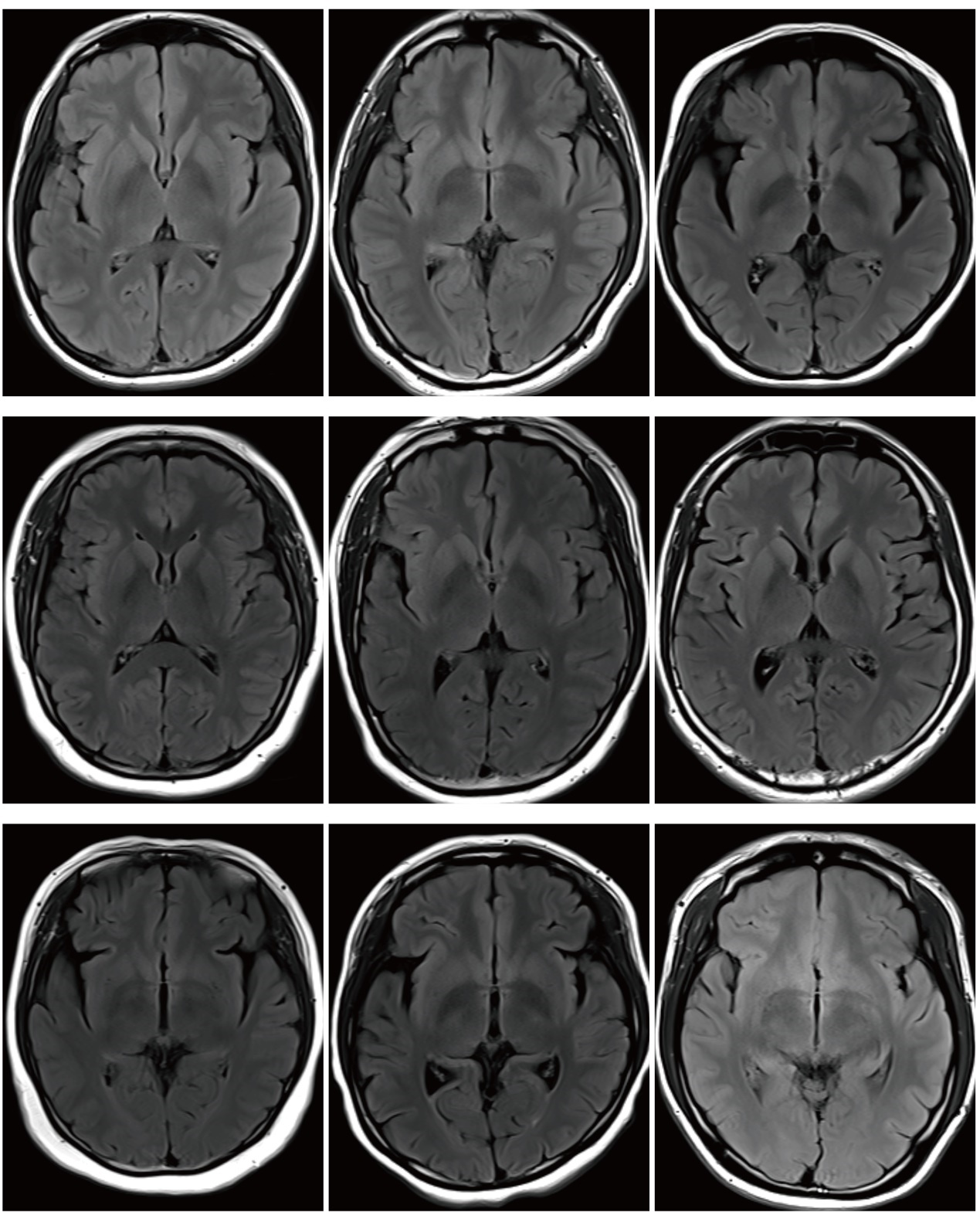
**

Supplementary Figure 14 No significant difference was observed in plasma Aβ40, Aβ42, NfL, and α-syn levels between preNIID and HCs in cohort 2. Mann-Whitney U test was used for comparison.


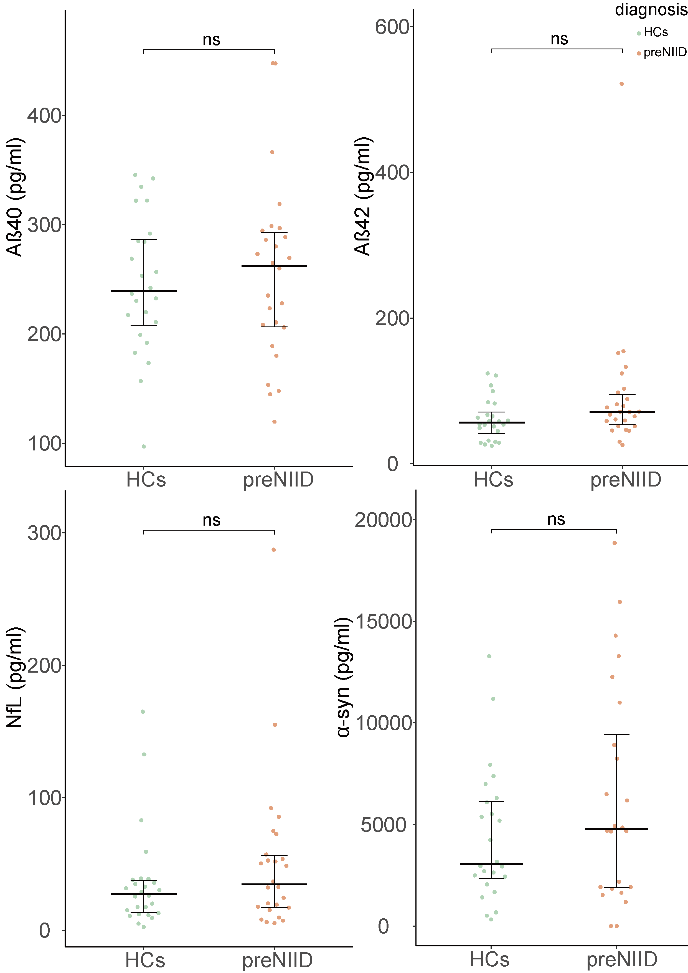


## Supplementary Figure 15 Comparison of p-tau/Aβ42 and Aβ42/40 ratios between HCs and preNIID individuals in cohort 2.

No statistical differences were observed between HCs (n = 26) and preNIID (n = 26). Mann-Whitney U test was used for comparison.


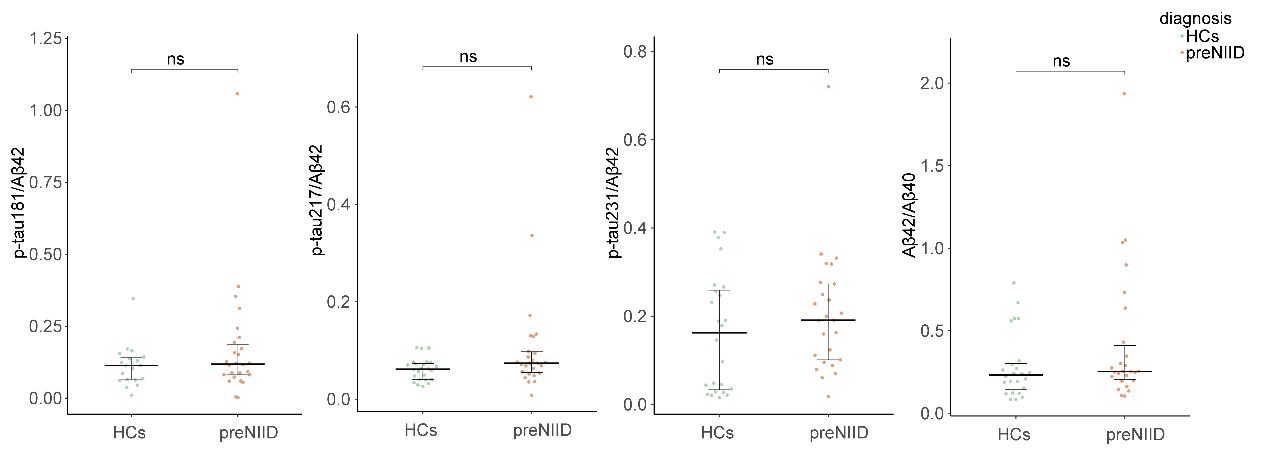


## Supplementary Table 4 Basic information of four phenotypes of NIID in cohort 1

|  | Muscle weakness-dominant subtype (n = 9) | Dementia-dominant subtype (n = 34) | Movement disorder-dominant subtype (n = 30) | Paroxysmal symptom-dominant subtype (n = 14) | p.overall | p.1 vs 2 | p.1 vs 3 | p.1 vs 4 | p.2 vs 3 | p.2 vs 4 | p.3 vs 4 |
| --- | --- | --- | --- | --- | --- | --- | --- | --- | --- | --- | --- |
| sex |  |  |  |  | 0.100 | 0.305 | 0.704 | 0.289 | 0.405 | 0.405 | 0.289 |
| female | 3 (33.3%) | 21 (61.8%) | 14 (46.7%) | 11 (78.6%) |  |  |  |  |  |  |  |
| male | 6 (66.7%) | 13 (38.2%) | 16 (53.3%) | 3 (21.4%) |  |  |  |  |  |  |  |
| age | 54.00 [32.00;64.00] | 66.00 [60.25;70.00] | 63.50 [56.50;65.00] | 63.00 [55.75;66.50] | 0.014 | 0.056 | 0.141 | 0.156 | 0.056 | 0.156 | 0.970 |
| AAO | 38.00 [29.00;64.00] | 60.00 [56.00;67.25] | 54.00 [46.50;59.00] | 60.00 [51.00;62.00] | 0.004 | 0.075 | 0.300 | 0.183 | 0.006 | 0.317 | 0.182 |
| duration | 7.00 [2.00;11.00] | 3.00 [2.25;6.75] | 7.00 [4.25;11.75] | 4.00 [2.25;5.00] | 0.021 | 0.631 | 0.631 | 0.509 | 0.023 | 0.723 | 0.023 |
| MMSE | 30.00 [27.50;30.00] | 16.00 [12.00;21.50] | 26.00 [22.00;28.00] | 26.00 [23.50;27.50] | <0.001 | <0.001 | 0.017 | 0.077 | <0.001 | <0.001 | 0.715 |
| Aβ40 | 268.27 [219.56;305.29] | 304.49 [261.40;376.39] | 324.83 [218.18;387.61] | 280.86 [230.02;313.05] | 0.318 | 0.415 | 0.464 | 0.901 | 0.901 | 0.415 | 0.415 |
| Aβ42 | 58.19 [53.93;89.47] | 67.42 [46.77;130.61] | 77.10 [48.00;124.51] | 51.87 [30.23;81.10] | 0.347 | 0.710 | 0.710 | 0.710 | 0.860 | 0.324 | 0.324 |
| NfL | 98.37 [39.03;525.82] | 93.09 [52.17;149.01] | 75.09 [34.54;125.48] | 48.45 [31.64;103.41] | 0.499 | 0.653 | 0.534 | 0.534 | 0.625 | 0.534 | 0.653 |
| α-syn | 7682.92 [2015.64;8383.90] | 6676.48 [4716.73;9025.87] | 5205.42 [2569.44;9720.25] | 7328.59 [3032.32;10701.49] | 0.899 | 0.946 | 0.946 | 0.946 | 0.946 | 0.946 | 0.946 |
| ptau181 | 8.80 [7.08;17.84] | 10.96 [7.96;19.95] | 9.59 [7.34;11.75] | 10.80 [8.41;18.30] | 0.152 | 0.424 | 0.816 | 0.372 | 0.208 | 0.816 | 0.208 |
| ptau217 | 4.40 [4.11;5.41] | 5.00 [4.25;7.38] | 5.97 [4.04;8.12] | 6.65 [4.47;9.82] | 0.511 | 0.578 | 0.578 | 0.578 | 0.578 | 0.578 | 0.578 |
| ptau231 | 14.14 [9.06;19.62] | 17.53 [11.84;31.47] | 13.51 [10.20;31.80] | 12.02 [7.56;32.75] | 0.896 | 0.954 | 0.954 | 0.954 | 0.954 | 0.954 | 0.954 |
| GFAP | 16.28 [13.88;19.98] | 31.12 [15.50;50.85] | 29.56 [13.00;38.11] | 11.94 [6.43;26.14] | 0.047 | 0.206 | 0.292 | 0.469 | 0.307 | 0.104 | 0.206 |
| GGC repeats | 109.00 [99.00;146.00] | 124.50 [109.25;137.25] | 124.00 [99.00;147.50] | 103.00 [88.00;135.00] | 0.404 | 0.988 | 0.988 | 0.458 | 0.988 | 0.458 | 0.458 |

Notes: p. overall the p value of overall comparison of these four groups (muscle weakness-dominant subtype, dementia-dominant subtype, movement disorder-dominant subtype, and paroxysmal symptom-dominant subtype) with Kruskal-Wallis H test; p.1 vs 2 (muscle weakness-dominant subtype vs dementia-dominant subtype), p.1 vs 3 (muscle weakness-dominant subtype vs movement disorder-dominant subtype), p.1 vs 4 (muscle weakness-dominant subtype vs paroxysmal symptom-dominant subtype), p.2 vs 3 (dementia-dominant subtype vs movement disorder-dominant subtype), p.2 vs 4 (dementia-dominant subtype vs paroxysmal symptom-dominant subtype), p.3 vs 4 (movement disorder-dominant subtype vs paroxysmal symptom-dominant subtype),were acquired through Benjamini & Hochberg adjustment method with Kruskal-Wallis H test for pairwise comparisons.

## Supplementary Table 5 Clinical information of ten patients with NIID completing tau-PET scan

| **Number** | **Age** | **Sex** | **Education** | **Subtype** | **Age at onset** | **GGC repeats** | **Chief complaint** | **MMSE** | **MoCA** |
| --- | --- | --- | --- | --- | --- | --- | --- | --- | --- |
| Patient 1 | 61 | Male | Middle school or above | Dementia | 58 | 10/127 | Episodic psychiatric and behavioral disturbances with concomitant cognitive decline persisting for 3 years. | 1 | 0 |
| Patient 2 | 67 | Female | Illiteracy | Dementia | 57 | 17/114 | Episodic headaches and memory decline for 10 years, progressive visual impairment for 1 year, with headache recurrence for 1 week | 10 | 2 |
| Patient 3 | 71 | Female | Middle school or above | Dementia | 65 | 10/96 | Recurrent episodes of vomiting and mental sluggishness over 6 years, with a recent recurrence persisting for over 10 days | 20 | 14 |
| Patient 4 | 66 | Female | Middle school or above | Dementia | 57 | 14/137 | Involuntary tremors involving the jaw and limbs for 9 years; urinary frequency and incontinence for 2 years; progressive memory decline for 2 years; generalised limb weakness for 2 months. | 21 | 13 |
| Patient 5 | 72 | Male | Middle school or above | Dementia | 71 | 22/104 | Progressive memory impairment for over 1 year and psychiatric abnormalities for 1 month. | 22 | 13 |
| Patient 6 | 63 | Female | Middle school or above | Dementia | 60 | 14/140 | Progressive memory decline accompanied by episodic speech arrest and vomiting for 3 years; generalised limb weakness for 2 years; urinary incontinence for 6 months. | 25 | 18 |
| Patient 7 | 66 | Female | Middle school or above | Paroxysmal symptom | 61 | 20/139 | Episodic impairment of consciousness for 5 years | 24 | 16 |
| Patient 8 | 57 | Male | Middle school or above | Movement disorder | 38 | 22/126 | Gradual onset of bilateral upper limb tremors over 9 years, with worsening and impaired mobility for the past 3 years. | 29 | 27 |
| Patient 9 | 51 | Female | Middle school or above | Muscle weakness | 43 | 23/95 | Chronic limbs numbness and weakness over 8 years, with progressive worsening accompanied by bradykinesia for 3 years; episodic vomiting for over 1 year. | 25 | 17 |
| Patient 10 | 61 | Male | Middle school or above | Movement disorder | 55 | 11/122 | Bilateral upper limb tremors persisting for 6 years, accompanied by generalised weakness and easy fatigability over 3 years, with progressive deterioration of symptoms in the past 6 months. | 25 | 27 |

# Reference

1. Jiao B, Ouyang Z, Liu Y, et al. Evaluating the diagnostic performance of six plasma biomarkers for Alzheimer's disease and other neurodegenerative dementias in a large Chinese cohort. *Alzheimers Res Ther* 2025; **17**(1): 71.
